# Investigation of cyanobacteria-hosted antibiotic resistance genes in cyanoHAB-impacted drinking water sources

**DOI:** 10.1007/s11356-026-37644-9

**Published:** 2026-03-31

**Authors:** Abigail Volk, Molly Mills, Soryong Chae, Jiyoung Lee

**Affiliations:** 1 Division of Environmental Health Sciences, College of Public Health, The Ohio State University, 1841 Neil Ave, Columbus, OH USA; 2 Environmental Sciences Graduate Program, The Ohio State University, Columbus, OH USA; 3 Department of Chemical and Environmental Engineering, University of Cincinnati, Cincinnati, OH USA; 4 Department of Food Science & Technology, The Ohio State University, Columbus, OH USA; 5 Infectious Diseases Institute, The Ohio State University, Columbus, OH USA

**Keywords:** Metagenomics, Microbiome, Resistome, Lake Erie, Grand Lake St. Marys, Harmful algal blooms, Cyanobacteria

## Abstract

Freshwater cyanobacterial blooms (cyanoHABs) are expanding across the world, and their frequency and severity are becoming more intense due to prevalent eutrophication and a changing climate. Traditionally, the concerns about cyanoHABs have mainly focused on cyanotoxins in water. CyanoHABs are also hypothesized to play a role in the antibiotic resistome, but whether cyanobacteria host clinically relevant antibiotic resistant genes (ARGs) in the environment is largely unknown. To investigate this emerging issue, we examined whether cyanobacteria host ARGs within the broader microbiome context. We looked for the presence of cyanobacteria-hosted ARGs using shotgun metagenomic sequencing of drinking water source samples collected during the bloom season (summer and fall) from Lake Erie and Grand Lake St. Marys (GLSM). ARGs were annotated using DeepARG and Resistance Gene Identifier (RGI). Cyanobacteria were annotated to host genes conferring putative antibiotic resistance, including efflux pumps *qac/EmrE*, *vatB*, *van genes*, and an OXA homolog. A maximum likelihood tree with cyanobacteria and OXA reference sequences showed OXA-like homology across multiple families of cyanobacteria. Most cyanobacteria sequences clustered in a large clade with *ybxI*, suggesting very limited or negligible class-D beta-lactamase activity, but a small subset formed a clade with OXA-2 and OXA-46. While those hits suggest potential resistance to clinical antibiotics, overall cyanobacteria were not found to host ARGs conferring resistance to drugs of last resort in these samples. Additionally, BLAST searches of the cyanobacteria ARG contigs and coding sequences resulted in top hits for cyanobacteria, further supporting that annotated genes are likely intrinsic rather than acquired. *rpoB2* and *arlR* ARG annotations appear to be spurious hits on housekeeping genes, which demonstrates the need to verify automated ARG annotation tool results. Selected cyanotoxins, cyanobacteria, and ARGs were also chosen for quantification. We found high levels of *Microcystis* in Lake Erie as well as *Planktothrix* and microcystin concentrations in GLSM, supporting previous trends in these water bodies. This study takes a novel approach, pairing the issues of cyanoHABs and ARGs together in two drinking water sources. In a changing climate, drinking water treatment strategies should consider the treatment and public health implications of multiple contaminants.

**Graphical Abstract:**

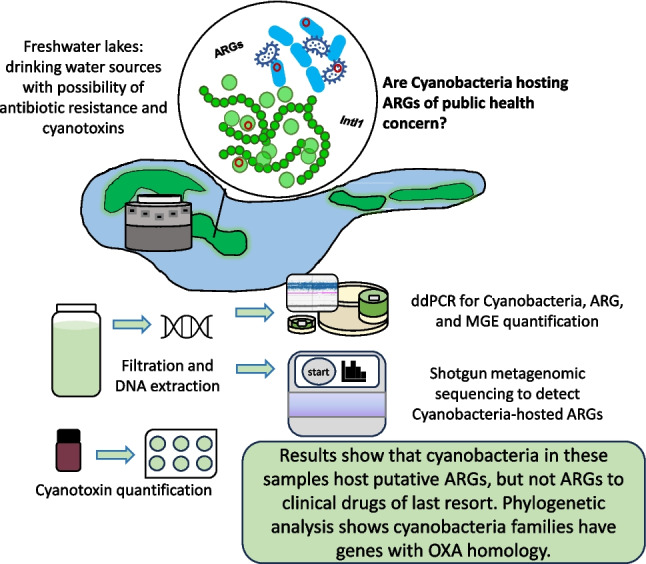

**Supplementary Information:**

The online version contains supplementary material available at 10.1007/s11356-026-37644-9.

## Introduction

Freshwater cyanobacterial harmful algal bloom (cyanoHAB) impacts range from ecosystem disruptions to public drinking water system closures (Jetoo et al. 2015). Cyanotoxins produced by freshwater bloom-forming cyanobacteria threaten human and animal health, causing gastrointestinal illness and hepatic toxicity (World Health Organization, 2020). Cyanotoxins also place an increased burden on drinking water treatment systems, requiring advanced treatment methods and heightened monitoring (Abbas et al. 2020; Collins Park Water Treatment Plant 2022; Henry 2015, p. 20). Two Ohio lakes with recurring toxic cyanoHABs are Lake Erie and Grand Lake St. Marys (GLSM). During the 2014 Toledo water crisis, microcystin concentrations in treated Lake Erie drinking water exceeded the World Health Organization (WHO) 1 ppb guideline because of *Microcystis*-dominated blooms (Jetoo et al. 2015; Steffen et al. 2017). GLSM is known for high microcystin concentrations and *Planktothrix* blooms (Jacquemin et al., 2023). Previous cyanoHAB research has primarily focused on the monitoring, ecology, and treatment of bloom-affected waters in Western Lake Erie (Jacquemin et al., 2023; Steffen et al. 2014a, b; Zhang et al. 2017). Both water bodies serve as drinking water sources and thus have great relevance to public health because of potential human and animal cyanotoxin exposure.

Parallel to the public health risk of cyanoHAB exposure is the threat of mobile and evolving antibiotic resistance (AR) in the environment (Larsson & Flach 2021). AR was first recognized as a clinical issue, creating treatment challenges in resistant human and animal infections. The antibiotic “resistome” includes all AR genes (ARGs) and ARG hosts—clinically relevant ARGs confer resistance to antibiotics used to treat infection, especially antibiotics of last resort. ARG hosts are not only pathogens, but also commensal bacteria and environmental microbes. Environmental matrices, including drinking water sources, serve as reservoirs of ARGs that may pass and evolve between bacteria (Kim & Cha 2021). Both AR and cyanoHABs are spurred by anthropogenic pollution and may intensify with changing climates (MacFadden et al. 2018; McGough et al. 2020; O’Neil et al. 2012; Reverter et al. 2020; Walsh et al. 2011; Y. Yang et al. 2019). CyanoHAB cyanotoxin risks in drinking water have been quantified, but AR risks in drinking water are more ambiguous. Identifying the presence of clinically concerning ARGs and their hosts in environmental reservoirs is critical to future work on environmental AR mobility and risk (Amarasiri et al. 2020; Sanganyado & Gwenzi 2019).

AR and cyanoHABs coincide in emerging research that suggests cyanobacteria might play a role in the resistome (Volk & Lee 2023). Cyanobacteria have been significantly correlated with ARGs using 16S rRNA sequencing and PCR quantification in network and redundancy analyses in a variety of matrices including water, biofilms, and fish guts (Gu et al. 2021; Hu et al. 2022; Jang et al. 2018; Matviichuk et al. 2022). However, those methods are associational and did not identify the ARG hosts. One study did detect *str*A-*str*B, *sul*1, qacΔE, and *intl*1 ARGs hosted in isolated strains of *Planktothrix* (Dias et al. 2019). The *str* and *sul*1 genes confer resistance to clinical antibiotics streptomycin and sulfonamide, respectively, and the *intI*1 gene is known to be a vehicle of ARG mobility. However, the study was limited to two isolated species of *Planktothrix* and not an environmental metagenome. Existing studies examine isolates and present associations between cyanobacteria and AR, but more research is needed to determine whether multiple cyanobacteria genera host ARGs within the environmental metagenome.

Consequently, our primary study objective is presence/absence identification of cyanobacteria-hosted ARGs in a subset of samples from CyanoHAB-impacted public drinking water sources. Using shotgun metagenomic sequencing allows for simultaneous detection of both ARGs and bacteria (Lee et al. 2020a, b; Quince et al. 2017). Lake Erie and GLSM lack extensive study of environmental AR (Ai et al. 2020; Mukherjee et al. 2021). Whether cyanobacteria in these two lakes serve as ARG reservoirs is largely unknown. Hence, we selected the drinking water intakes in these two lakes as sample locations. We hypothesized that cyanobacteria in these lakes would be reservoirs of clinically relevant ARGs because of historically high nutrient levels and agricultural fecal contamination. Previous work shows fecal pollution and nitrate to increase ARG abundance, supporting a general relationship between poor water quality, cyanoHABs, and AR (Hoorman et al. 2008; Karkman et al. 2019; Steffen et al. 2014a, b; Wang et al. 2024; Zhang et al. 2025).

A second objective is the employment of viable methods for the concurrent quantification of multiple potential public health concerns. Because the samples in this study are from raw lake water sourced for human consumption, we quantified cyanotoxin concentration, several target cyanobacteria and ARGs, and the presence and relative abundance of other microbes. The coexistence of cyanobacteria, cyanotoxins, and ARGs in drinking source waters is infrequently studied in parallel, especially in these cyanoHAB locales, and it is expected that advanced drinking water treatments reduce cyanobacteria and cyanotoxins to drinking water standards. Historically, cyanoHABs have been investigated from a nutrient standpoint. As understanding of the microbiome has grown, placing cyanoHABs in the context of the broader microbial community is important for designing future experiments investigating how other microbes might interact with and influence cyanoHABs (Pound et al. 2021). We hypothesized high relative abundance of *Microcystis* in Lake Erie and *Planktothrix* in GLSM because of previous cyanoHAB trends in these water bodies, and we also report on the broader microbial community.

The recurring cyanoHABs that have shut down the public water supply in these locations warrant continued monitoring of the concentration and presence of contaminants. This study reports on cyanotoxins and cyanobacteria while examining cyanobacteria-hosted ARGs in the natural environment.

## Materials and methods

### Sample collection, water filtration, and DNA extraction

Surface water grab samples were collected adjacent to the Lake Erie Toledo Collins Park Water Treatment Plant intake crib (approximately 41.7 N 83.4 W, coordinates rounded) on May 15, July 19, and September 16, 2022. Grab samples were collected from the GLSM Celina Water Treatment Plant water intake line (approximately 40.540 N 84.568 W, coordinates rounded) on June 27, August 16, and October 14, 2022. Sampling dates captured the bloom season, which generally occurs from July through October. A sampling event also took place in May for Lake Erie and June for Grand Lake St. Marys, before the usual peak bloom season. Sample information is summarized in Supplementary Information 1 (Table S1). Representative images from both locations were taken with a field microscope (ioLight, Southampton, England, UK). Water samples were vacuum filtered through sterile 0.2 µm PC membranes (100–200 mL of water) as technical replicates, and the filtration volume was recorded. DNA was extracted from the membranes using a DNeasy PowerSoil Pro kit (Qiagen, Valencia, CA, USA) according to the manufacturer’s instructions. To lyse the membrane, a Bead Ruptor12 (Omni International, Kennesaw, GA, USA) was used for 3-min cycles × 3 at max speed (5 m/s). DNA concentration and quality were measured using both a Qubit Fluorometer (Thermo Fisher Scientific, Waltham, Massachusetts) and NanoDrop™ 2000 Spectrophotometer (Thermo Fisher Scientific, Waltham, Massachusetts). Extracted DNA was stored at −20 °C.

### Cyanotoxin quantification and droplet digital PCR

Water samples from each collection date were stored in glass vials at −20 °C for determining cyanotoxin concentration using cyanotoxin ELISA. A freeze–thaw process was repeated three times to rupture intact cyanobacterial cells and release intracellular toxins so that total cyanotoxin concentrations could be measured (Greenstein et al. 2021; Ohio EPA, 2021). ELISA test kits (Gold Standard Diagnostics, Warminster, PA, USA) were used to detect total congener-independent microcystins (PN520011 from EPA Method 546), anatoxins (PN520060), and saxitoxins (PN52255B), according to the manufacturer’s instructions. If the concentrations measured above the ELISA cyanotoxin test kit threshold, samples were diluted (between 1:2 and 1:10).

DropletDigital™ PCR (ddPCR) was performed using the QX200™ system (Bio-Rad, Hercules, CA, USA). ddPCR assays were used to quantify cyanobacteria and ARGs as previously described (Ai et al. 2020). The following ddPCR assays were run: total *Microcystis* marker gene from the phycocyanin intergenic spacer (PC-IGS) (Kurmayer & Kutzenberger 2003), microcystin toxin-producing gene *mcyE Microcystis* (Sipari et al. 2010), microcystin toxin-producing gene *mcyE Planktothrix* (Ngwa et al. 2014), anatoxin-producing gene *anaC* (Rantala-Ylinen et al. 2011), and saxitoxin-producing gene *sxtA* (Murray et al. 2011). These cyanobacterial genes are referred to as PC-IGS, *mcyE Microcystis*, *mcyE Planktothrix*, *anaC*, and *sxtA*, respectively. ddPCR assays were performed for the mobile genetic element *intI1* (MGE) (González-Plaza et al. 2019), and for the ARGs sulfonamide resistance (*sul1*) and tetracycline resistance (*tetQ*) (Luo et al. 2010). These genes were selected because of widespread tetracycline and sulfonamide antibiotic use in healthcare and agriculture (Baran et al. 2011; Daghrir & Drogui 2013) and MGE as a transporter of ARGs (Ghaly & Gillings 2021).

ddPCR droplets were generated using the QX200 droplet generator (Bio-Rad), and thermal cycling was conducted with the C1000 Touch™ Thermal Cycler (Bio-Rad, Hercules, CA, USA). Gene copies were quantified using the QX200 Droplet Reader and QuantaSoft Software (Bio-Rad, Hercules, CA, USA), and a positive and negative control were included with every run. Final gene concentrations were calculated after accounting for DNA template volume, DNA extraction elution volume, and volume of water filtered for each sample. Reaction reagents, conditions, and primer information for each assay are provided in the Supplementary Information (Table S2). Technical replicates were performed for both cyanotoxin and ddPCR.

### Shotgun metagenomic sequencing

One water sample from each collection date was selected for shotgun metagenomic sequencing based on quality and concentration. DNA was diluted to 2 ng/µL and sent to the Applied Microbiology Services Laboratory (AMSL) at The Ohio State University for DNA library generation and shotgun metagenomic sequencing. Forty nanograms of total DNA was used to generate libraries using Illumina’s DNA Prep (M) Tagmentation (Illumina Inc.). Library sizing, quality, and concentration were checked with the BioAnalyzer High Sensitivity DNA Kit (Agilent Technologies, Santa Clara, CA, USA), qPCR (ProNex NGS Library Quant Kit, Promega, Madison, WI, USA), and Qubit (Thermo Fisher Scientific, Waltham, Massachusetts), respectively. Libraries were sequenced at 2× 150 cycles on an NextSeq 2000 (Illumina, San Diego, CA, USA).

### Bioinformatics

Raw sequencing output files were processed using the Ohio Supercomputer Center (Ohio Supercomputer Center 1987). Sequencing data can be accessed using the accession number PRJNA1124395 on the National Center for Biotechnology Information (NCBI) Sequence Read Archive (SRA) (https://www.ncbi.nlm.nih.gov/sra/PRJNA1124395). Average sequencing depth was 68,564,321 raw reads (stdev = 18,126,829 reads). Adapters were removed and raw paired reads were cleaned and trimmed using BBDuk from BBTools v38.69 (BBDuk Guide 2014). Quality checks were compared for raw read pairs, adapter trimmed read pairs, and fully cleaned read pairs for each sample using FastQC v0.11.8 (Andrews 2010). MultiQC v1.7 (Ewels et al. 2016) was used to visualize the FastQC output. Raw reads were assembled using MegaHit v1.2.8 (D. Li et al. 2015) using the default K-mer list. Sequencing information (number of reads and number of base pairs) is generated using SeqKit (Shen et al. 2016) and is provided in Supplementary Information (Table S3). The final assembled contigs from each sample were annotated for taxonomy using Contig Annotation Tool (CAT) v5.2.3 (von Meijenfeldt et al. 2019). To annotate contigs, CAT uses Prodigal v2.6.3 to find genes (Hyatt et al. 2010), Diamond version 2.0.6 to perform protein alignment (Buchfink et al. 2015), and the NCBI non-redundant protein database (nr) to add taxonomy to annotated contigs. The microbial community was filtered from the complete CAT annotations by filtering for all bacteria and archaea, as well as select eukaryotes (Table S4). The CAT annotations were used for relative abundance calculations from kingdom to species.

The assembled contigs were sorted into genome bins and assessed for quality. The MetaWRAP (Alneberg et al. 2014; Kang et al. 2019; Uritskiy et al. 2018; Wu et al. 2016) pipeline was used for both binning assembled contigs into genomes and assessing quality. Dereplication was performed with dRep (Olm et al. 2017) at 99% average nucleotide identity. Medium and high-quality bins (≥ 70% completion, < 10% contamination; ≥ 95% completion < 5% contamination, respectively) were retained as metagenome assembled genomes (MAGs) using CheckM (Parks et al. 2015). The genome bins are referred to as metagenome-assembled genomes (MAGs). The MAGs were taxonomically classified with GTDB-Tk (Chaumeil et al. 2020).

ARG counts were normalized to the Giga base pair (Gbp) depth of each sequencing sample’s assembly, resulting in gene copies per Gbp (GC/Gbp). The final assembled contigs from each sample were annotated with the ARG deep-learning model DeepARG-LS using the default high-confidence settings (Arango-Argoty et al. 2018). DeepARG was selected because it uses a more environmental and less clinical definition of ARG to reduce the false negative rate associated with stricter tools. This is helpful for environmental AR because DeepARG helps serve to identify potential ARGs and genes that may be important in ARG evolution, not just strictly acquired ARGs (Arango-Argoty et al. 2018). “High confidence ARGs” annotated by DeepARG are referred to as such (*probability* > 0.8, alignment identity > 50, alignment e-value < 1e-10) (Arango-Argoty et al. 2018). However, because these cutoffs can discard ARGs, lower confidence ARGs (annotated below these thresholds) on cyanobacteria are also reported and clearly marked as “possible” ARGs.

The Comprehensive Antibiotic Resistance Database (CARD) Resistance Gene Identifier (RGI) was used as a second tool with default settings for annotating ARGs (Alcock et al. 2020). For metagenomics, CARD is the most frequently used ARG database and relies on an expertly curated database (de Abreu et al. 2021). RGI was also used to annotate ARGs on MAGs. RGI results are displayed for perfect and strict hits only (perfect means predicted gene 100% matches known ARG at the amino acid level; strict means sequences are not exact matches, but the genes are likely functional and greater than the manually curated bit score for each ARG). The outputted ARG predictions were paired to CAT taxonomic annotations using the contig ID of each sequence for ARG host analysis. MAGs annotated as cyanobacteria with an annotated RGI ARG were then submitted to ResFinder, the strictest strict tool, to check for high-confidence, acquired ARGs (> 90% ID, > 60% minimum length) (Florensa et al. 2022). MAGs identified as cyanobacteria were also annotated with Mobile Element Finder (Johansson et al. 2021).

Three select ARGs annotated on cyanobacterial contigs were further checked using NCBI BLASTn (Johnson et al. 2008; Sayers et al. 2024) to confirm contig taxonomic annotation. Cyanobacterial contigs also underwent an initial BLASTx to check alignment to conserved ARG domains. Finally, BLASTp was used for specific genes identified by NCBI ORFfinder (Table 2). Based on this review, the OXA annotation was selected for further phylogenetic analysis because of its potential public health relevance. Open reading frames for the OXA-annotated contig were predicted with ORFfinder. The translated amino acid was then searched via BLASTp to confirm conserved domain, as well as in HHPred (Söding et al. 2005) and Phyre2 (Kelley et al. 2015) to confirm OXA homology. The literature was reviewed for other OXA phylogenetic studies. In Lupo et al., a phylogenetic cluster with cyanobacterial (as well as Alphaproteobacteria, Betaproteobacteria, and Gammaproteobacteria) taxonomy and the OXA-2, OXA-20, and OXA-46 genes was reported previously (Lupo et al. 2022). As a result, a PSI-BLAST against NCBI RefSeq limited to cyanobacteria (taxid 1117) was performed with the sequence of interest and reference genes *Bacillus subtilis ybxI* (NP_388091.1), *Pseudomonas aeruginosa* OXA-46 (AFP97030.1), *Acinetobacter baumannii* OXA-20 (ACC55538), and multi-species OXA-2 (WP_001007673). The results were filtered to include sequences with coverage > 80% and identity > 35% (185 sequences). Near identical sequences were removed from the results using CD-HIT (Fu et al. 2012; W. Li & Godzik 2006) to cluster at 95% identity, resulting in 132 clusters. These 132 sequences and the 5 PSI-BLAST initial query sequences were aligned using MAFFT (Katoh & Standley 2013) and trimmed using trimAl (Capella-Gutiérrez et al. 2009). IQ-TREE (Nguyen et al. 2015) was used to generate the maximum likelihood tree using ModelFinder Plus and 1000 ultrafast bootstrap replicates (-m MFP -bb 1000). The tree was then visualized in FigTree (*FigTree*, n.d.), with the OXA-20 representative sequence shown at the root as the outgroup.

### Statistical analyses and visualization

Data cleaning, statistics, and visualization were performed in R v4.1.1. To compare target gene concentrations by location, the non-parametric Wilcoxon rank-sum test was used. Pairwise Spearman correlations were performed for the full set of ddPCR data. The average ddPCR gene concentration and average microcystin concentration from each sample date were standardized using the mean and standard deviation before running a principal component analysis (PCA). An ordinary least squares regression between *mcy*E *Planktothrix* concentration and microcystin was performed.

Phyloseq v1.38.0 (McMurdie & Holmes 2013) was used for microbial community statistical analysis. To obtain alpha and beta diversity, CAT microbial community taxonomic annotation counts were standardized by rarefying to even depth (311,586 counts using the rarefy_even_depth function in phyloseq), which was the smallest library size of the 6 samples, as we did not want to drop any samples from analysis (McKnight et al. 2019; Weiss et al. 2017). Phyloseq and VEGAN v2.6.4 were used to obtain Shannon indices for α-diversity and Bray–Curtis dissimilarity for β-diversity (Dixon 2003). Cyanobacteria were filtered from the CAT microbial results, and the same methods were performed to standardize and compute Shannon index and Bray–Curtis dissimilarity of the cyanobacterial communities. To test differences in metadata by β-diversity, permutational multivariate analysis of variance (PERMANOVA) using Bray–Curtis dissimilarity was performed using the VEGAN adonis2 function (Dixon 2003). β-diversity was visualized using non-metric multi-dimensional scaling (NMDS) plots. The Tidyverse v1.3.2 and ggplot2 were used for visualization (Wickham et al. 2019).

## Results

### Cyanobacteria, ARGs, and toxin concentrations in water sources by location

**Fig. 1:**
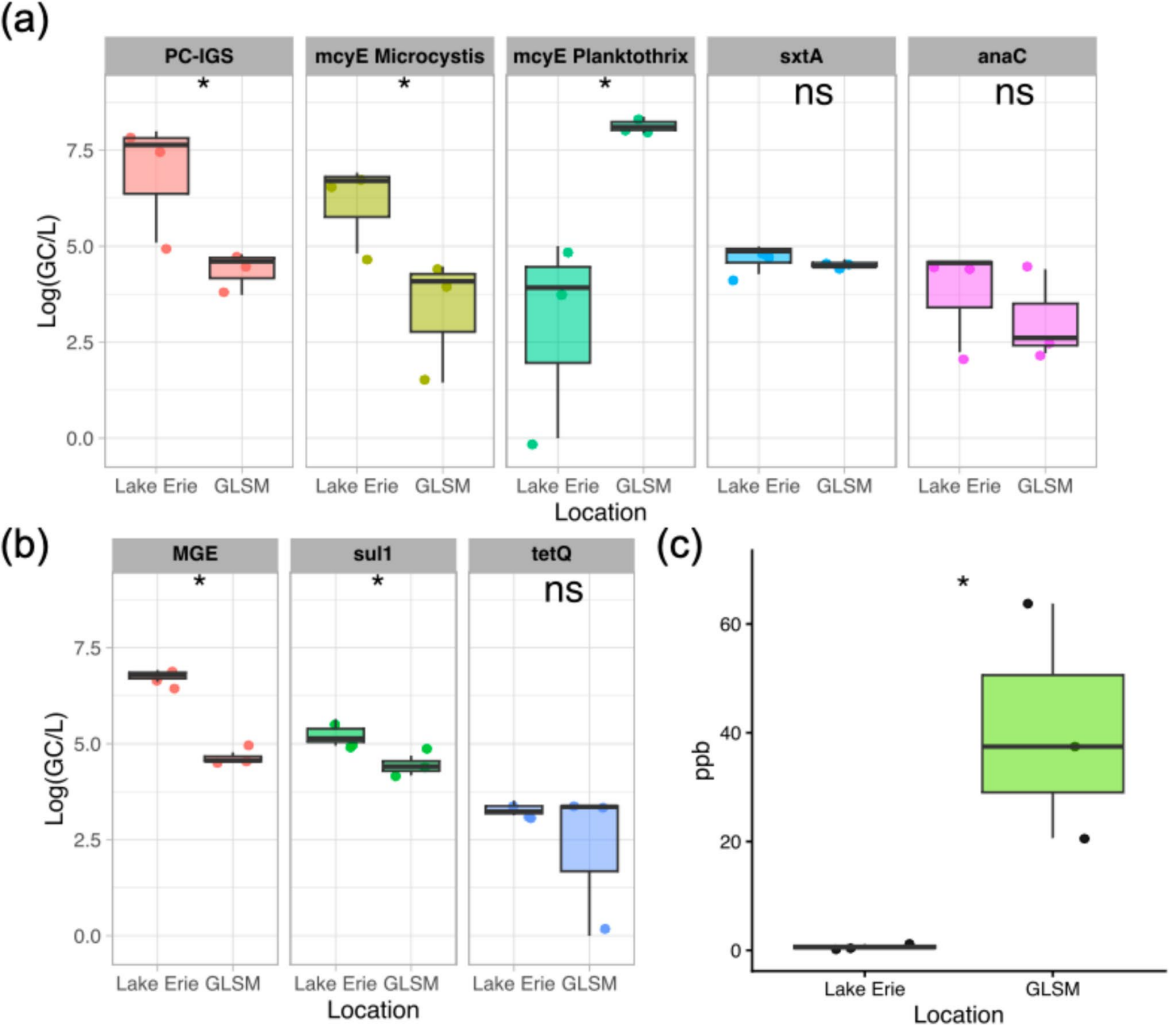
Average concentrations of cyanobacteria, toxic cyanobacteria, ARGs, and microcystin by location. **a** Cyanobacteria and toxin-producing cyanobacteria measured by ddPCR, **b** ARGs measured by ddPCR, and **c** microcystin ELISA concentrations. Wilcoxon rank sum test was performed. * = *p* < 0.1, ns = not significant

All cyanobacteria and ARGs targeted using ddPCR were quantified in both Lake Erie and GLSM (Fig. 1a). Total *Microcystis* concentrations, measured with PC-IGS, were significantly higher in Lake Erie than GLSM (*p* < 0.1). *mcy*E *Microcystis* (microcystin-producing *Microcystis)* concentrations were also significantly higher in Lake Erie (*p* < 0.1). *mcy*E *Planktothrix* (microcystin-producing *Planktothrix)* concentrations were significantly higher in GLSM (*p* < 0.1). *sxtA* (saxitoxin-producing cyanobacteria) and *anaC* (anatoxin-producing cyanobacteria) were detected in both locations, but there were no significant differences by location. *sul*1 and MGE concentrations were significantly higher in Lake Erie (*p* < 0.1) (Fig. 1b). There was no significant difference in *tet*Q by location.

**Fig. 2:**
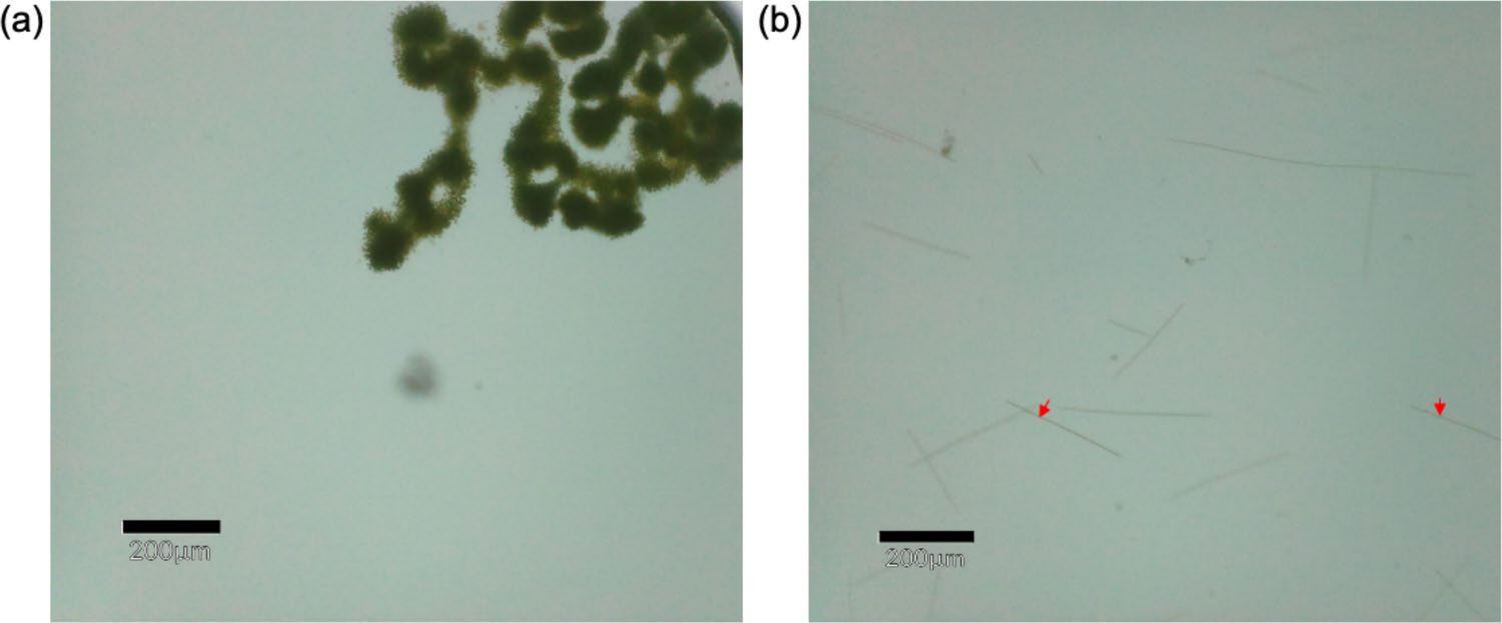
Microscope images. **a** Lake Erie samples taken in July showing *Microcystis* spp. colony morphology. **b** Grand Lake St. Marys sample taken in August showing *Planktothrix* spp. morphology

Through cyanotoxin ELISA testing, microcystin (MC) concentration was significantly higher in GLSM (*p* < 0.1) (Fig. 1c). While saxitoxin and anatoxin-producing cyanobacteria were detected in both locations, these cyanotoxins were below the limit of detection using the respective ELISA kits. Representative microscopic images taken from Lake Erie samples show colonies consistent with the morphology of colony-forming *Microcystis* spp. (Fig. 2a), while representative images taken from Grand Lake St. Marys samples show filamentous morphology consistent with *Planktothrix* spp. (Fig. 2b).

### Relationships between concentrations of cyanobacteria, ARGs, and microcystins

**Fig. 3:**
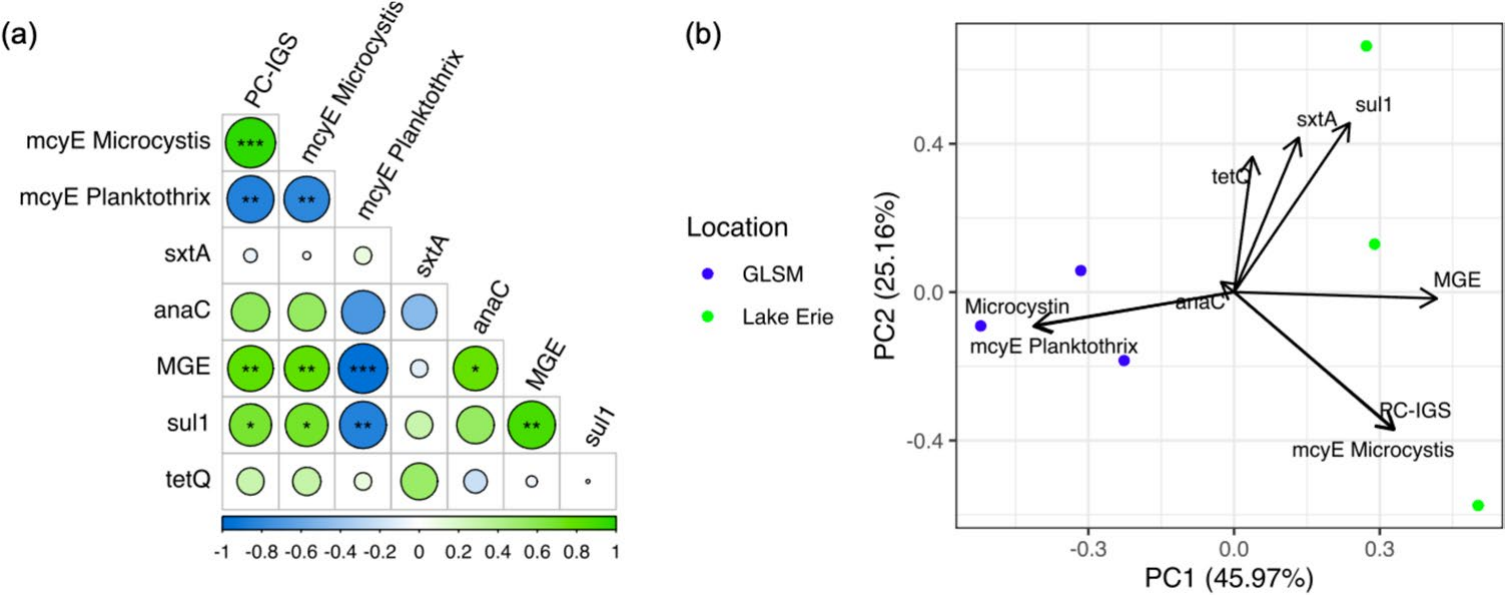
Association between measured variables. **a** Correlations between concentrations of cyanobacteria and ARGs. *** = *p* < 0.001, ** = *p* < 0.01, * =  < 0.05. **b** PCA for cyanobacteria, ARGs and microcystin concentrations. 45.97% of the variability is explained by PC1, and 25.16% of the variability is explained by PC2

Multiple pairwise Spearman’s correlations were statistically significant between cyanobacteria and ARG concentrations quantified by ddPCR (Fig. 3a). PC-IGS and *mcy*E *Microcystis* were positively correlated (*p* < 0.001*, ρ* = 0.95), while *mcy*E *Planktothrix* was negatively correlated with PC-IGS (*p* < 0.01*, ρ* = −0.77) and *mcy*E *Microcystis* (*p* < 0.01*, ρ* = −0.73). There were also positive correlations between MGE and PC-IGS (*p* < 0.01*, ρ* = 0.72), *mcy*E *Microcystis* (*p* < 0.05*, ρ* = 0.66), and *sul1* (*p* < 0.01*, ρ* = 0.81). Scatterplots of significant pairwise variables are summarized in Figure S1 (see Supplementary Information).

A PCA was done to reduce dimensionality of all the variables measured with ddPCR with microcystin (Fig. 3b). Principal component 1 (PC1) explains 45.97% of the variance in the tested variables, and principal component 2 (PC2) explains 25.16% of the variance. Loadings of each variable are shown in Table S5 (see Supplementary Information). GLSM samples and Lake Erie samples are divided across PC1. *mcy*E *Planktothrix* concentration had moderate negative loadings on PC1, consistent with GLSM samples, while MGE, PC-IGS, and *mcyE Microcystis* concentrations had moderate positive loadings on PC1 consistent with Lake Erie samples. The PCA shows a correlation between microcystin and *mcyE Planktothrix* concentrations, as well as a correlation between PC-IGS and *mcyE Microcystis*. An ordinary least squares (OLS) regression was fit between *mcyE Planktothrix* and microcystin concentrations (microcystin = 0.48 + 2.6e-07 * *mcyE Planktothrix* concentration, *p* < 0.001) (Figure S2, Table S6). An important limitation is that the small sample size restricts robust correlation conclusions and makes adjusting correlations by location impossible.

### Microbial community

**Fig. 4:**
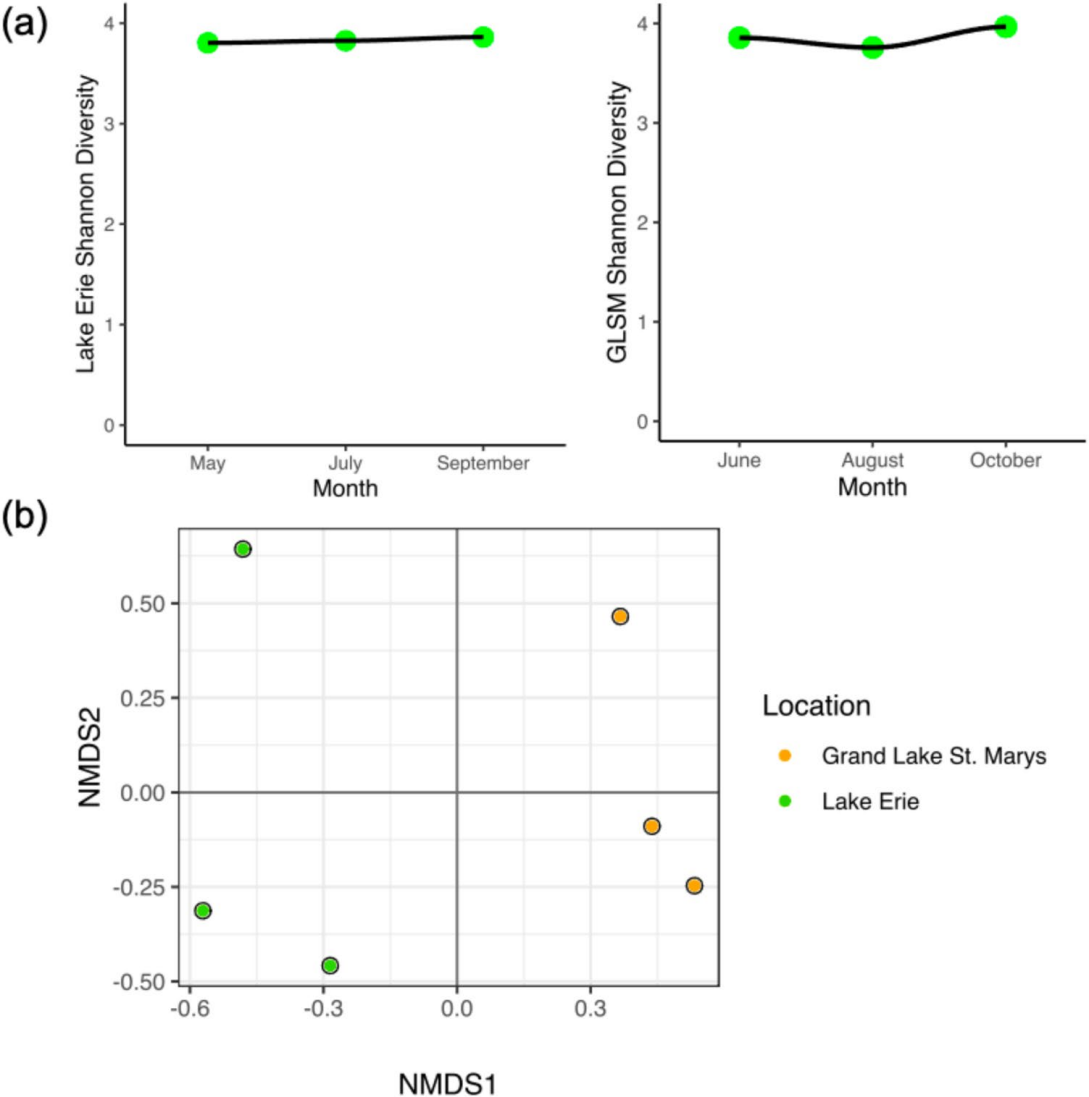
Microbial community. **a** α-Diversity of the microbial community for Lake Erie (top left) and GLSM (bottom left) over the measured months, using the Shannon index. **b** NMDS plot showing Bray–Curtis dissimilarity for β-diversity of the microbial communities between the two locations

Microbial community Shannon indices were consistent across all samples, ranging from 3.72 to 4.00 (Fig. 4a). Shannon indices did not differ statistically by location (*p* > 0.05). The microbial communities of both locations were dominated by bacteria (> 98% relative abundance, based on CAT contig annotations) (Figure S4). Eukaryota had average relative abundances of 1.5% and 0.3% in Lake Erie and GLSM, respectively. Archaea had relative abundances of less than 0.4% at both locations. There were not significant differences in the microbial β-diversity by sample location (*p* = 0.2) (Fig. 4b). Though not significant, GLSM and Lake Erie samples ordinate separately across NMDS1. There were no significant differences in microbial β-diversity with any of the cyanobacteria and ARGs concentrations, or with microcystins concentration (PERMANOVA). Those with a *p*-value less than 0.2 were microcystin concentration (*p* = 0.10), MGE (*p* = 0.11), *sul1* (*p* = 0.11), and *mcyE Planktothrix* (*p* = 0.11).

Average relative abundances were calculated using CAT annotations for microscopic Eukaryota and Bacteria and are reported in Figure S4. The average relative abundances for bacteria at a phyla level are reported in Figure S6. The top two phyla annotated for both locations were Actinobacteria (Lake Erie 35.2%, GLSM 32.2%) and Proteobacteria (Lake Erie 25.7%, GLSM 28.1%). Cyanobacteria had an average relative abundance of 11.6% in Lake Erie and 6.5% in GLSM.

### Cyanobacterial community

**Fig. 5:**
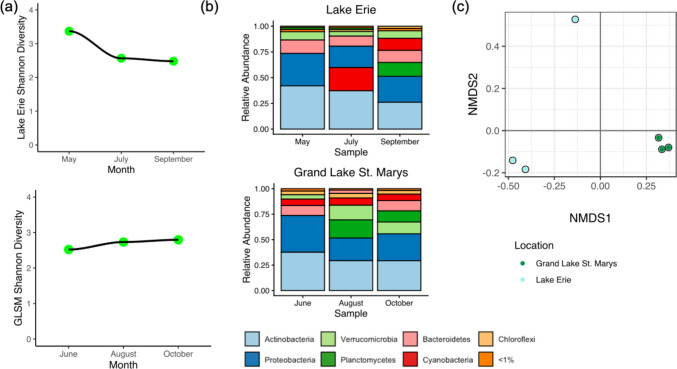
Cyanobacterial community. **a** α-Diversity of the cyanobacterial community for Lake Erie (top left) and GLSM (top right) over the measured months, using the Shannon index. **b** Cyanobacteria genera average relative abundance for the two locations, by month. **c** NMDS plot showing Bray–Curtis dissimilarity for β-diversity of the cyanobacterial communities for the two locations

Cyanobacterial Shannon indices were consistent across all samples, ranging from 2.48 to 2.80, except for a peak in Lake Erie in May of 3.37 (Fig. 5a). The average Shannon index did not differ statistically by location. The average relative abundances of cyanobacteria genera varied by month and location (Fig. 5b). On average, GLSM was dominated by *Planktothrix* (90.0%) followed by *Aphanizomenon* (2.3%). Lake Erie’s top genus was *Microcystis* (47.2%) followed by *Aphanizomenon* (22.4%), *Synechococcus* (8.0%), *Planktothrix* (6.8%), *Pseudanabaena* (6.0%), and *Cyanobium* (4.0%).

There were significant differences in the cyanobacterial β-diversity with MGE (*p* < 0.05), PC-IGS (*p* < 0.01), and *Microcystis mcyE* (*p* < 0.01) (PERMANOVA). Though β-diversity was not significant with location (*p* = 0.1) at the cyanobacterial level, GLSM and Lake Erie samples ordinate across NMDS1 (Fig. 5c). GLSM samples cluster tightly on the NMDS plot, while there is a large spread between the Lake Erie May sample and the other 2 months. Cyanobacterial β-diversity was not significant with microcystin (*p* = 0.13) or *Planktothrix mcyE* (*p* = 0.11) (PERMANOVA).

From May to July in Lake Erie, the cyanobacterial portion of the community switches from an *Aphanizomenon*/*Planktothrix* mixture to *Microcystis-*dominant. Of the small amount of *Microcystis* spp. annotated in May, 91.7% were *Microcystis wesenbergii* (Figure S9). However, by July, the major *Microcystis* species observed is *Microcystis aeruginosa* (74.8% of *Microcystis* annotations). At GLSM, the relative abundances of *Planktothrix* spp. remain constant throughout the season (Figure S10). The composition of *Planktothrix* spp. includes *Planktothrix agardhii* (~40.0% of *Planktothrix* spp.), *Planktothrix tepida* (~33% of *Planktothrix* spp.), *Planktothrix paucivesiculata* (~12% of *Planktothrix* spp.), and *Planktothrix prolifica* and *rubescens* (both ~5% of *Planktothrix* spp.) (Figure S11). This matches the high and consistent *mcyE Planktothrix* gene copies observed (Figure S3d). Microcystin levels appear to increase throughout the season (Figure S3c).

### Resistome

There was a total of 722 ARG annotations on contigs from the two tools over the 6 samples: 239 high-confidence annotations from DeepARG and 483 strict annotations from RGI. Of these, only 32 contigs received annotations from both tools, 8 of which were the same or similar genes (Table S6) and 24 of which were not (Table S7), suggesting different annotations by the tools used. This is likely due to the distinct algorithms and aims of the two tools, which is why both were used for ARG annotation. The genes in agreement between both tools were AAC(6’)-II, *qac*, and FOS genes. Genes annotated by both tools but not in agreement are as follows: *adeF* annotated by RGI was annotated as *MexF* by DeepARG, *qacG* and *qacJ* annotated by RGI were annotated as *EmrE* or *AbeS* by DeepARG, and *rpsL* annotated by RGI was annotated as *rpoB2* by DeepARG.

**Fig. 6:**
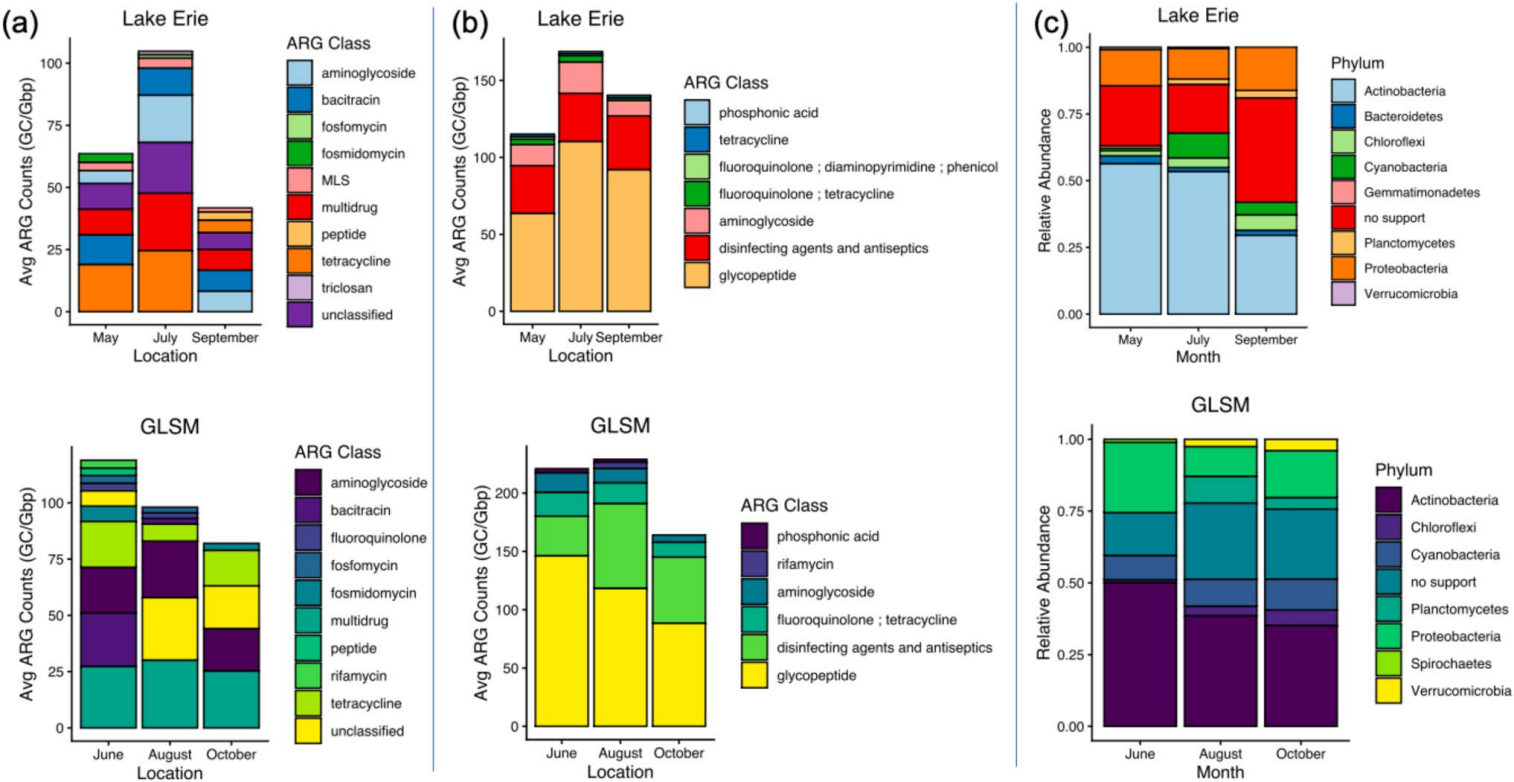
ARG results from shotgun metagenomic sequencing. Average ARG class abundance for contigs by location using **a** DeepARG for annotation by location and month and **b** RGI for annotation by location and month. **c** ARG host average relative abundance by location of month. The results of the two tools were merged. No support indicates there was not an annotation at a phyla level

The abundance (GC/Gbp) of high-confidence and strict ARGs was not significantly different by location for either tool (DeepARG *p* = 0.4; RGI *p* = 0.2. Wilcoxon rank-sum test). The top five classes of ARG for DeepARG for both locations, in no order, were multidrug, tetracycline, aminoglycoside, bacitracin, and unclassified (Figure S13a). The top four classes of ARG for RGI were glycopeptide, disinfecting agents and antiseptics, “fluoroquinolone; tetracycline”, and “fluoroquinolone; diaminopyrimidine; phenicol” (Figure S13b). Composition of the major ARG classes generally stayed consistent for each month in both locations by tool. Abundance of ARGs was highest during summer (July for Lake Erie for both tools; June for GLSM using DeepARG; and August for GLSM using RGI), although this was not significant (Fig. 6a and b).

After merging results from both tools (high confidence from DeepARG and strict for RGI) and removing duplicate hits, the relative abundance of ARG host phyla by month and location was calculated (Fig. 6c). The top three hosts by average relative abundance at a phyla-level annotation were Actinobacteria (41% Lake Erie; 46% GLSM), Proteobacteria (17% Lake Erie; 14% GLSM), and cyanobacteria (10% Lake Erie; 5% GLSM). However, a large portion of hosts was not annotated to a phyla-level (22% Lake Erie; 27% GLSM) (Table S8).

### Cyanobacteria were annotated as ARG hosts and contain insertion sequences

Cyanobacteria MAGs and contigs were annotated to host ARGs by both tools (Table 1). In Lake Erie, the cyanobacteria contigs *Microcystis*, *Pseudanabaena*, *Vulcanococcus,* and *Cyanobium* were annotated as hosts, including the MAG *Microcystis aeruginosa* with *vanY*. The only cyanobacterial host annotated to a genus level in GLSM was *Planktothrix*, including the MAG *Planktothrix agardhii*, which was annotated with *vanH*, *vanY*, *vanT*, and *qacJ*. These hosts match the dominant cyanobacteria genera in each location. The high-confidence cyanobacterial ARGs annotated by DeepARG were *arlR*, *vatB*, and *rpoB2*, while the strict ARGs annotated by RGI were *qac* and *van* genes. The “possible” DeepARG output (exploratory, less stringent ARG identification cutoffs) was also matched to host. Interestingly, *Synechococcus* and *Cyanobium* in Lake Erie were annotated to host ARGs OXA and *EmrE*, respectively (Table S9). No cyanobacteria MAGs hosted high-confidence, acquired ARGs using the ResFinder tool.

**Table 1:** All cyanobacterial contigs and MAGs annotated with ARGs by DeepARG (high confidence) and RGI

Sample ID	Tool	Read	Read type	Gene	ARG class	Bitscore	Identity	Host Genus	GC/GBP
LakeErie1	DeepARG	k99_299602	contig	*arlR*	Unclassified	73.6	50		1.72
GLSM2	DeepARG	k99_55634	contig	*arlR*	Unclassified	85.9	52.6	*Planktothrix*	3.40
GLSM2	DeepARG	k99_103113	contig	*rpob2*	Multidrug	1166.4	54.2		3.40
GLSM2	RGI	k99_332416	contig	*vanW* gene in *vanI* cluster	Glycopeptide	75.9	35.09		3.40
GLSM2	RGI	k99_200543	contig	*vanY* gene in *vanG* cluster	Glycopeptide	72	28.97		3.40
GLSM2	RGI	bin 2	MAG	*vanH* gene in *vanB* cluster	Glycopeptide		39.93	*Planktothrix agardhii*	
				*qacJ*	Disinfecting agents and antiseptics		39.8		
				*vanY* gene in *vanM* cluster	Glycopeptide		34.35		
				*vanT* gene in *vanG* cluster	Glycopeptide		34.76		
LakeErie3	DeepARG	k99_1079718	contig	*arlR*	Unclassified	82	50	*Vulcanococcus*	1.36
LakeErie3	DeepARG	k99_1358475	contig	*arlR*	Unclassified	117.5	50	*Microcystis*	1.36
LakeErie3	DeepARG	k99_752142	contig	*vatB*	Mls	223.4	54.2	*Pseudanabaena*	1.36
LakeErie3	DeepARG	k99_952662	contig	*rpoB2*	Multidrug	1155.6	54.2		1.36
LakeErie3	RGI	k99_497629	contig	*vanY* gene in *vanA* cluster	Glycopeptide	74.7	34.26		1.36
LakeErie3	RGI	k99_806870	contig	*vanY* gene in *vanF* cluster	Glycopeptide	50.8	24.81	*Cyanobium*	1.36
LakeErie3	RGI	k99_1271941	contig	*vanT* gene in *vanG* cluster	Glycopeptide	189.1	39.03		1.36
LakeErie3	RGI	k99_904564	contig	*vanY* gene in *vanA* cluster	Glycopeptide	69.7	32.86	*Microcystis*	1.36
LakeErie3	RGI	k99_814031	contig	*vanY* gene in *vanA* cluster	Glycopeptide	64.7	39.51	*Microcystis*	1.36
LakeErie3	RGI	k99_968253	contig	*vanY* gene in *vanG* cluster	Glycopeptide	53.9	25.95		1.36
LakeErie3	RGI	k99_968985	contig	vanW gene in vanG cluster	Glycopeptide	76.6	31.45		1.36
LakeErie3	RGI	k99_1218399	contig	*vanT* gene in *vanG* cluster	Glycopeptide	223	37.13		1.36
LakeErie3	RGI	k99_356549	contig	*vanT* gene in *vanG* cluster	Glycopeptide	211.1	37.13		1.36
LakeErie3	RGI	k99_1436454	contig	*vanT* gene in *vanG* cluster	Glycopeptide	218.8	36.86		1.36
LakeErie3	RGI	k99_578325	contig	*vanY* gene in *vanM* cluster	Glycopeptide	66.6	32.06	*Microcystis*	1.36
LakeErie3	RGI	k99_578405	contig	*vanY* gene in *vanM* cluster	Glycopeptide	81.3	34.33	*Microcystis*	1.36
LakeErie3	RGI	k99_1228262	contig	*vanY* gene in *vanB* cluster	Glycopeptide	71.2	35.07	*Pseudanabaena*	1.36
LakeErie3	RGI	k99_1136471	contig	*vanY* gene in *vanM* cluster	Glycopeptide	64.3	41.98	*Microcystis*	1.36
GLSM4	DeepARG	k99_679575	contig	*arlR*	Unclassified	85.9	52.6	*Planktothrix*	2.51
GLSM4	DeepARG	k99_306726	contig	*arlR*	Unclassified	84.3	50		2.51
GLSM4	DeepARG	k99_494758	contig	*rpoB2*	Multidrug	1166.4	54.2		2.51
GLSM4	RGI	k99_404057	contig	*vanY* gene in *vanM* cluster	Glycopeptide	72.4	34.35	*Planktothrix*	2.51
GLSM4	RGI	k99_319526	contig	*vanW* gene in *vanI* cluster	Glycopeptide	75.9	35.09		2.51
GLSM4	RGI	k99_204074	contig	*vanT* gene in *vanG* cluster	Glycopeptide	209.5	34.76	*Planktothrix*	2.51
GLSM4	RGI	k99_31865	contig	*vanH* gene in *vanB* cluster	Glycopeptide	202.2	39.93	*Planktothrix*	2.51
GLSM4	RGI	k99_481554	contig	*qacG*	Disinfecting agents and antiseptics	77	40		2.51
GLSM4	RGI	k99_166359	contig	*vanT* gene in *vanG* cluster	Glycopeptide	252.3	39.14		2.51
GLSM4	RGI	k99_311848	contig	*qacJ*	Disinfecting agents and antiseptics	89.4	39.8		2.51
GLSM4	RGI	k99_414578	contig	*vanY* gene in *vanG* cluster	Glycopeptide	68.9	31.58		2.51
LakeErie5	RGI	k99_867127	contig	*vanY* gene in *vanB* cluster	Glycopeptide	62	30.25		1.67
LakeErie5	RGI	k99_956387	contig	*qacG*	Disinfecting agents and antiseptics	79.3	40.22	*Cyanobium*	1.67
LakeErie5	RGI	k99_314657	contig	*vanT* gene in *vanG* cluster	Glycopeptide	218.8	36.86		1.67
LakeErie5	RGI	bin 7	MAG	*vanY* gene in *vanM* cluster	Glycopeptide		34.33	*Microcystis aeruginosa*	
				*vanY* gene in *vanA* cluster	Glycopeptide		39.51		
GLSM6	DeepARG	k99_113113	contig	*arlR*	Unclassified	85.5	52.6	*Planktothrix*	3.15
GLSM6	DeepARG	k99_247612	contig	*rpoB2*	Multidrug	1166.4	54.2		3.15
GLSM6	RGI	k99_170654	contig	*vanT* gene in *vanG* cluster	Glycopeptide	209.5	34.76	*Planktothrix*	3.15
GLSM6	RGI	k99_396426	contig	*qacJ*	Disinfecting agents and antiseptics	89.4	39.8		3.15
GLSM6	RGI	k99_532018	contig	*vanY* gene in *vanM* cluster	Glycopeptide	72.4	34.35	*Planktothrix*	3.15
GLSM6	RGI	k99_306830	contig	*vanY* gene in *vanG* cluster	Glycopeptide	72.4	29.66		3.15
GLSM6	RGI	k99_421242	contig	*vanH* gene in *vanB* cluster	Glycopeptide	202.2	39.93		3.15
GLSM6	RGI	k99_309285	contig	*vanW* gene in *vanI* cluster	Glycopeptide	75.9	35.09		3.15

Insertion sequences were annotated on cyanobacteria bins using Mobile Element Finder. Ten insertion sequences were annotated on *Planktothrix agardhii* bins, one was annotated on the *Cyanobium* bin, and twenty-nine were annotated on the *Microcystis aeruginosa* bin (Table S13). Insertion sequence identities ranged from 0.73 to 1 while coverage ranged from 0.10 to 0.96.

To provide further context on the ARG annotations, one of each type of ARG annotated on cyanobacteria hosts was verified further using NCBI BLAST (Table S12). BLASTn results for a contig with a DeepARG annotation for *arlR* were 98.6% identity to a section of a previously sequenced *Microcystis aeruginosa* complete genome containing a response regulator transcription factor. Similarly, BLASTn for a contig containing the rpoB2 annotation was 97% identity for previously sequenced *Microcystis viridis* NIES-102, containing the RNA polymerase beta subunit. The possible *EmrE* annotation resulted in a 91.1% identity hit for *Cyanobium gracile* PCC 6307 putative flavoprotein. The RGI *vanY* annotation had 100% coverage and identity to previously sequenced *Planktothrix agardhii* M15 family metallopeptidases, and the RGI *qacJ* annotation has 100% coverage and identity to a previously sequenced *Planktothrix* multidrug efflux small multidrug resistance transporter. The results for the *vatB* annotation resulted in 89.8% identity to *Pseudanabaena* sp. ABRG5-3 chloramphenicol O-acetyltransferase. BLASTn for the contig containing the possible DeepARG OXA annotation interestingly was both 92.2% identity to the *Synechococcus* sp. RS9909 chromosome containing the penicillin binding transpeptidase domain protein, a class-D beta-lactamase. Based on the BLAST results, three cyanobacteria ARG hits (OXA, *vatB*, and *qacG*) were analyzed further to identify conserved domains (Table 2). The top BLAST hits for both the specific gene and the contig for these selected sequences were cyanobacteria taxonomy and conserved domains supported the putative ARG annotations.

**Table 2:** Top BLASTn, BLASTx, and BLASTp hit results for select cyanobacteria ARG contigs and genes

Original annotation	Tool	Contig ID	BLAST method	Top Hit Description	Hit Scientific Name	Query Cover	E value	Per. Ident	Accession	BLASTx Conserved Domain	Conserved Domain Accession
OXA	DeepARG “possible”	k99_1036155	contig, BLASTn core_nt	*Synechococcus* sp. RS9909 chromosome, complete genome	*Synechococcus* sp.	94	0	92.24	CP047943.1		
			contig, BLASTx RefSeq	Class D beta-lactamase [*Vulcanococcus* sp.]	*Vulcanococcus* sp.	54	6e-176	96.69	WP_296447350.1	YbxI	COG2602
			gene, BLASTp RefSeq	class D beta-lactamase [Vulcanococcus sp.]	*Vulcanococcus* sp.	95	0	96.69	WP_296447350.1		
*vatB*	DeepARG "high confidence"	k99_752142	contig, BLASTn core_nt	MAG: *Microcoleus* sp. isolate GLPA chromosome	*Microcoleus* sp.	52	0	89.38	CP181292.1		
			contig, BLASTx RefSeq	TetR/AcrR family transcriptional regulator [*Pseudanabaena* sp. UWO311]	*Pseudanabaena* sp. UWO311	42	6e-122	95.29	WP_142653398	LbH_XAT AcrR TetR_C_28	cd03349 pfam17937 COG1309
			gene, BLASTp RefSeq (first ORF)	Vat family streptogramin A O-acetyltransferase [*Pseudanabaena* sp. UWO310]	*Pseudanabaena* sp. UWO310	99	5e-84	95.35	WP_142599971		
			gene, BLASTp RefSeq (second ORF)	TetR/AcrR family transcriptional regulator [Pseudanabaena sp. UWO311]	*Pseudanabaena* sp. UWO311	100	3e-126	95.29	WP_142653398.1		
*qacG*	RGI	k99_481554	contig, BLASTn core_nt	MAG: *Aphanizomenon flos-aquae* KM1D3_PB chromosome, complete genome	*Aphanizomenon flos-aquae* KM1D3_PB	99	3e-137	95.53	CP051528.1		
			contig, BLASTx RefSeq	DMT family transporter [*Aphanizomenon* sp. CS-733/32]	*Aphanizomenon* sp. CS-733/32	100	2e-52	99.05	WP_271776363.1	Multi_Drug_Res	pfam00893
			gene, BLASTp RefSeq	DMT family transporter [*Aphanizomenon* sp. CS-733/32]	*Aphanizomenon* sp. CS-733/32	100	1e-48	98.82	WP_271776363.1		

### Cyanobacteria contain homologous OXA-like domains

A total of 185 cyanobacteria sequences with moderate identity (35–75%) to the 5 sequence OXA-like query (contig from this study, OXA-2, OXA-20, OXA-46, and *ybxI*) resulted from PSI-BLAST. Of the 132 clusters retained for the maximum likelihood tree, most of the sequences clustered in a large clade with *YbxI*. However, 11 cyanobacteria sequences formed a smaller clade with the OXA-2 and OXA-46 reference sequences.

## Discussion

### Lake Erie and GLSM are dominated by different cyanobacterial genera

One study objective was to report on the cyanobacterial community. The results of this study support historical trends, observations, and hypotheses for Lake Erie. Lake Erie is known for seasonal succession of *Microcystis* species, particularly microcystin-producing *Microcystis aeruginosa* (Ouellette et al. 2006). As evidenced in our study, the primary cyanobacteria species in July and September was *Microcystis aeruginosa* (Figure S9). Microcystin-producing *Microcystis (mcyE Microcystis)* and microcystins (Fig. 1) were detected as well, indicating that some of the *Microcystis aeruginosa* detected likely produced microcystin. Microcystins were detected in Lake Erie at less than 1.5 ppb. While the guideline for drinking water post-treatment is < 1 ppb, microcystin concentrations in Lake Erie have been measured at wide ranges, from < 1 ppb to maximums over 1000 ppb in previous years (Steffen et al. 2014a, b). The apparent cyanobacterial community shift in Lake Erie from May to July is also supported by previous work. *Microcystis* in May was barely detectable and a small proportion of the cyanobacteria genera—the May *Microcystis* species were the majority *Microcystis wesenbergii* (Figure S8a/9a), which is generally known to be non-toxic (Komárek 2016). Warmer temperatures, decreases in pH, toxin production, decreased grazing by protozoa and other plankton, and colony formation may contribute to the dominance of *Microcystis* spp. (Ladds et al. 2021; Wilhelm et al. 2020; Xiao et al. 2018).

Another observation supporting historical trends in Lake Erie is the annotation of other previously identified cyanobacteria taxa alongside *Microcystis* including *Aphanizomenon, Planktothrix*, *Cyanobium*, *Synechococcus*, and *Pseudanabaena* (Fig. 5b) (Berry et al. 2017; Ladds et al. 2021; Steffen et al. 2014a, b). *Pseudanabaena* and *Aphanizomenon* explain the ddPCR detection of saxitoxin- and anatoxin-producing cyanobacteria as they are known producers of these toxins that occur with high relative abundance in Lake Erie (Cegłowska et al. 2022; Cirés & Ballot 2016; Lyra et al. 2001). However, we did not detect saxitoxins or anatoxins, suggesting synthesis of these toxins was not occurring, or occurring at concentrations too low to detect. We detected *Cyanobium* and *Synechococcus*, which are sometimes overlooked in Lake Erie because they are harder to observe and not regarded as threats, in contrast to genera like *Microcystis* (Steffen et al. 2014a, b; Wilhelm et al. 2006). Our results show the benefit of using shotgun metagenomic sequencing to detect lesser-described genera in Lake Erie.

The results for GLSM also corroborate previous work. There was very little variation over the months sampled at GLSM, possibly because the first sample date was in June when temperatures were warm and the bloom season was already underway. Similar to previous literature on GLSM, the dominant cyanobacteria genus was *Planktothrix* (Steffen et al. 2012; Steffen et al. 2014a, b) (Figure S10), a contrast to Lake Erie. Microscope imagery shows filamentous-type morphology in GLSM (Fig. 2b). *Planktothrix* filaments are long, slender floss-like shapes which are composed of hundreds of cells (Kurmayer et al. 2016). Of annotated GLSM *Planktothrix* species (Figure S11), *Planktothrix agardhii*,* Planktothrix rubescens*, and *Planktothrix* UBA8407 have been reported to produce microcystin (Gaget et al. 2015; Kurmayer et al. 2004; Zhang et al. 2020). Moderate microcystin levels were detected (> 15 ppb) (Figure S3c), consistent with species annotations, high microcystin-producing *Planktothrix (Planktothrix mcyE)* concentrations, and previous detection of microcystin above the WHO’s contact advisory level in GLSM (Jacquemin et al., 2023).

Overall, *Microcystis* dominated Lake Erie and *Planktothrix* dominated GLSM, while GLSM samples had higher microcystin concentrations. Identifying cyanobacteria genera and toxin concentration is important for water treatment, as treatments vary in effectiveness and high toxin concentrations by the drinking water intake cause concern (He et al. 2016).

### Across locations, the total microbial community contained freshwater bacteria but sample size limited power for statistical comparison

Another study objective was to report microbial community relative abundances. The bacterial phyla in both Lake Erie and GLSM consisted mainly of Actinobacteria, Proteobacteria, Bacteroidetes, Verrucomicrobia, Planctomycetes, Cyanobacteria, and Chloroflexi (Fig. 5b). These are all common freshwater bacterial phyla, and it is expected that at high taxonomic levels (phyla), they remain reasonably consistent from water body to water body (Hazarika & Thakur 2020; Kaboré et al. 2020; Zwart et al. 2002).

All statistical tests of the total microbial community β-diversity with other variables were not significant (*p* > 0.05). However, the samples from the two locations repeatedly clustered separately: on either side of PC1 for the cyanobacterial/toxin concentrations PCA, and on either side of NMDS1 for both the microbial and cyanobacterial communities (Figs. 4c and 5c). The sample size limited the power for statistical tests of microbial community β-diversity with location and other covariates (Kelly et al. 2015), but we still include the results here. Statistical differences could be seen for the cyanobacterial community β-diversity with MGE, total *Microcystis* (PC-IGS), and *mcyE Microcystis* (*p* < 0.1). We are interested in the association of MGE with cyanobacteria because MGE is associated with greater gene mobility and higher contamination (Bennett 2008; Ghaly & Gillings 2021; Gillings et al. 2015). Future studies should include a sample design robust to further investigating that association. Our findings support recent suggestions of reporting the broader microbial community in cyanobacteria-focused studies. In a 2021 opinion piece, cyanobacteria researchers proposed a paradigm shift where heterotrophic bacteria and the broader microbial community are reported together (Pound et al. 2021).

Our main objective was identifying the presence/absence of cyanobacteria-hosted ARGs, though we report on the microbial community, cyanotoxins, and cyanobacteria and ARG ddPCR quantification. A recognized limitation of this study for interpreting statistical results is the sample size. As such, statistics should be taken cautiously, especially pairwise correlations and PERMANOVAs where we could not account for location or season. Limited power is likely why β-diversity was not significant with location for the microbial community. PERMANOVA performed with small sample sizes (five samples per group) was previously shown to have less power to detect differences in simulated distance matrices than larger sample sizes (10 or 20 samples per group) (Kelly et al. 2015). Therefore, there may be statistical differences in the microbial community by site, but more power is needed to detect them. However, targeted ddPCR and ELISA methods did show differences by location*,* showing the benefits of pairing targeted methods with non-targeted methods such as shotgun sequencing. Large multivariate shotgun datasets are generally less sensitive to differences than targeted methods (Goodrich et al. 2014). Seasonality likely contributes to changes in these microbial communities, as evidenced by the NMDS plot (Fig. 4b), which displays each sample month. Microbial communities, like most communities in ecology, can show seasonal and temporal trends (Faust et al. 2015; Gilbert et al. 2012). May Lake Erie and June GLSM separate from the rest of the samples over NMDS2, supporting existing research showing seasonal variations impact environmental microbiome composition (Fierer 2017; Neu et al. 2021), though more robust sampling is needed to look more specifically into seasonal microbial community changes at these sites.

### The resistome generally lacked pathogens and ARGs of clinical concern

There is extensive research on cyanoHABs in Lake Erie and GLSM, but significantly less on AR (Ai et al. 2020; Lee et al., 2023; Mukherjee et al. 2021). Therefore, we included reporting on the resistomes as a study objective. There were differences in the ARG annotations between the two tools. This speaks to differences in databases, algorithms, and tool objectives. CARD attempts to identify validated and curated ARGs conservatively using an alignment approach, while the predictive DeepARG deep-learning model is suited to reduce the false negative rate of classic alignment approaches for detecting novel ARGs (Arango-Argoty et al. 2018; Davis et al. 2023; Papp & Solymosi, 2022). By using both, we intended to annotate a wide variety of ARGs. There were eight annotations that were highly similar between the two tools, implying some agreement (Table S6).

However, *adeF* annotated by RGI was annotated as *MexF* by DeepARG. The bit score was too low to be classified as *MexF* by RGI. This is detailed in research on *adeF* and *MexF* efflux pump classification challenges (Yao & Yiu, 2019). When the bit score does not meet the CARD *MexF* classification threshold, the gene is usually classified as *adeF*. *qacG* and *qacJ* annotated by RGI were annotated as *EmrE* or *AbeS* by DeepARG. *EmrE* is often a hit for *qac* searches, as both are efflux pumps (Wassenaar et al., 2015). *AbeS* is also an efflux pump, which could explain the discrepancy (Srinivasan et al., 2009) (Supplementary Information 1; Table S7).

The watersheds surrounding both Lake Erie and GLSM are used for agricultural purposes (Hoorman et al. 2008; Michalak et al., 2013), and therefore, ARGs relevant to agricultural antibiotics and disinfectants were expected. Tetracycline and sulfonamide resistance genes are regularly detected in the environment because of widespread historic and current use in veterinary and agricultural settings (Cadena et al., 2018; Guo et al., 2014). The tetracycline resistance gene *tetQ* was detected using ddPCR, and tetracycline resistance genes were annotated in the top five ARG classes in both sample locations using DeepARG, with similar abundance, though *tetQ* was not annotated (Fig. 6). There were RGI hits for tetracycline ARGs in Lake Erie. The sulfonamide resistance gene sul1 was also detected in both locations via ddPCR, but not detected in the metagenomic sequencing using RGI or DeepARG. This is likely because of a limitation of the DeepARG-LS model, which does not perform well annotating specifically sulfonamide and mupirocin AR gene classes but performs well for other classes of ARG (Arango-Argoty et al. 2018). Kasugamycin resistance genes were detected in the top five ARG classes at both locations for DeepARG. Kasugamycin is used in plant pest control, a highly agricultural purpose (McGhee & Sundin, 2011). Largely, the antibiotics that the ARGs in these samples confer resistance to are first-generation antibiotics, not drugs of last resort such as carbapenems or colistin (Miłobedzka et al., 2022).

Relative abundances of high-confidence ARG host phyla generally matched the most abundant bacterial phyla in the microbial community (Fig. 6 and Figure S6). The high-confidence ARG annotations show no conclusive evidence of potential human pathogens, though because sequencing is DNA-based there is some uncertainty (Hirsch et al., 2010). One limitation of shotgun sequencing is that it is highly reliant on existing databases and predictive models, and not all contigs receive taxonomic or functional gene annotations. Many contigs annotated as ARG hosts did not have genus or species-level taxonomic classifications, limiting certainty in pathogenic host presence/absence. Nonetheless, Betaproteobacteria were annotated as high-confidence ARG hosts in all samples, and *Burkholderiaceae* was annotated as a high-confidence ARG host in the May Lake Erie sample. These families can contain opportunistic and primary pathogens such as *Burkholderia pseudomallei* and *Burkholderia mallei* (Coenye, 2014; Ferro et al., 2019). *Planctomycetes* have been identified as a possible human opportunistic pathogen in a case study that reported identification of *Planctomycetes* DNA in the blood of two immunocompromised patients (Drancourt et al., 2014). *Planctomycetes* was annotated to be hosting efflux pump ARGs in GLSM in August and October and in Lake Erie in July. Previous literature has reported efflux pumps in the *Planctomycetes* genome (Cayrou et al., 2010). Finally, *Pseudomonadaceae* was annotated with *rsmA* in May in Lake Erie. Some members of this family are opportunistic pathogens, such as *Pseudomonas aeruginosa*, though we did not have annotation to a species level (Iglewski, 1996). One study demonstrated that *rsmA*, a virulence-related gene, is linked to increased AR in *P. aeruginosa* (Mulcahy et al., 2006). Overall, we conclude that while there is uncertainty about potential pathogenic ARG hosts using DNA-based methods, there is also not an alarming presence of species-level pathogenic AR bacteria in this dataset.

Resistomes in different environmental matrices (as well as living hosts) exist on a continuum (Pal et al., 2016; Zhuang et al., 2021). There are different levels of clinical relevance based on the presence of mobile or clinically relevant ARGS, particularly when hosted by potential pathogens or commensals (Kim & Cha 2021). An environmental AR conceptual continuum summarizes a few main variables when estimating resistome clinical relevance: the exposure probability of that resistome to humans (low versus high); the presence of resistance genes to antibiotics of clinical last resort; the presence and relative abundance of MGEs; and whether AR bacteria are pathogens or commensals (Miłobedzka et al., 2022). We detected MGE *intI1* using ddPCR—but the drinking water sources in this study receive advanced water treatment before humans consume it, and we generally did not detect substantial clinically relevant ARGs or pathogenic bacteria. Therefore, the resistome in these drinking water sources sits on the lower risk end of the continuum and is more “environmental” when compared to clinically relevant resistomes. Wastewater treatment plant influent, effluent, hospital discharge, urban storm water, and direct agricultural runoff often have more concentrated antibiotic, pathogen, and fecal contamination than many environmental water sources, which are linked to their resistomes (Kim & Cha 2021; Pal et al., 2016). A past study found lake water generally has less annotated ARGs of concern than wastewater (Karkman et al., 2018). Simultaneously, antibiotic resistance is recognized as an ancient and innate mechanism in environmental bacteria (D’Costa et al., 2011). Hence, resistomes are natural and existed before anthropogenic pressures, which makes distinguishing innate environmental resistomes from those of greater public health relevance challenging (Wright, 2010). In summary, these waters are relatively uncontaminated with ARGs compared to other matrices like wastewater effluent.

### Cyanobacteria were annotated as ARG Hosts

The main objective was examination of cyanobacteria-hosted ARGs. In this study, cyanobacteria were annotated as ARG hosts using both tools, but outstanding questions include whether ARGs hosted by cyanobacteria are likely to confer resistance, whether the ARGs are intrinsic or acquired, and whether they have public health relevance. The cyanobacterial genera hosting ARGs generally matched predominant cyanobacterial genera in the respective locations. We had hypothesized that a community with a high relative abundance of cyanobacteria would also have a high abundance of ARGs harbored by cyanobacteria, given that the resistome can be driven by the microbial composition (Baquero et al. 2013; Qiao et al. 2021; Song et al. 2017).

#### ARG tool outputs should be examined carefully

The DeepARG “high confidence” genes annotated with cyanobacteria hosts included *rpoB2*,* arlR*, and *vatB* (Table 1). *rpoB2* is a mutation of the RNA polymerase β-subunit gene analyzed in *Nocardia* spp. conferring resistance to rifampin (Ishikawa et al. 2006; Wehrli 1983). Rifampin antibiotics are used for mycobacterial, *Clostridium difficile*, and Gram-positive infections, including tuberculosis (Beloor Suresh et al. 2022). While not a traditional ARG, the *rpoB2* mutation has been reported in multiple environmental matrices, such as sediments, hospital wastewater, and global sludges (Imchen & Kumavath 2021; Manoharan et al. 2021; Xu et al. 2022; Z. Yang et al. 2022). One review suggested that *rpoB2* annotations can be false hits in shotgun datasets because the ARG is a single nucleotide polymorphism (SNP) of the *rpoB* housekeeping gene (Davis et al. 2023). The authors, therefore, caution against assuming this is an ARG. *Microcystis* spp. are known to contain the gene *rpoB* (Pound & Wilhelm 2020). Our BLAST result, which aligned the rpoB2 contig with the rpoB gene in the *Microcystis* genome, therefore suggests that this is likely not a “true” ARG hit. Similarly, *arlR* is a gene identified to regulate a multidrug transporter in *S. aureus* (Fournier et al. 2000) and was annotated on many contigs within our dataset, including cyanobacteria. Davis et al. (2023) cautions against assigning genes regulating efflux pumps to ARGs, as regulators are sometimes removed before analysis of AR datasets (Davis et al. 2023; K. Lee et al. 2020a, b). Our BLAST results indicate that this is a regulator innate to the cyanobacteria that is unlikely to regulate an ARG. The genes we annotated that appear to be spurious hits are often reported as “bona fide” ARGs in other studies without any further verification, which demonstrates that ARG annotation tools should be interpreted cautiously for genes that may be homologous to other essential genes in bacteria.

#### ARGs analyzed in this study are likely intrinsic

All hits that were confirmed with BLAST searches (Table 2, Table S12) resulted in top results with high identity and coverage to cyanobacteria. In Table 2, both the taxonomy from BLASTn of the contig and BLASTp/BLASTx of the gene are shown to be cyanobacteria, providing support that these genes are likely intrinsic rather than acquired.

*vatB* is an acetyltransferase discovered on plasmids in *Staphylococcus aureus* that inactivates streptogramin (Allignet & el Solh 1995). In our samples, it was annotated on *Pseudanabaena* and was 89.8% BLASTn identity to the *Pseudanabaena* sp. ABRG5-3 chloramphenicol O-acetyltransferase gene. It was also 86.3% identity to *Pseudanabaena* with a Vat family streptogramin annotation. RGI hits annotated on cyanobacteria include *van* and *qac* genes. The *qacJ* annotation on *Planktothrix agardhii* resulted in a 100% coverage and identity BLAST hits for small multidrug resistant efflux pumps on *Planktothrix*. Previously, the *qac* resistance gene *qac∆E* was detected performing PCR on *Planktothrix* strains (Dias et al. 2019). Various *van* annotations make up most cyanobacteria ARG hits. One reason for this is that cyanobacteria are Gram-negative, and Gram-negative bacteria are not affected by glycopeptide antibiotics such as vancomycin because it cannot cross the outer membrane (Antonoplis et al. 2019). We therefore suggest that these glycopeptide hits are the result of innate resistance.

#### Multiple cyanobacteria families have class-D beta-lactamase homology

The unique DeepARG “possible” ARGs annotated on cyanobacterial contigs were *OXA* and *EmrE*, using more exploratory thresholds (Table S9). There are hundreds of existing *OXA* genes, which are β-lactamases and can include carbapenemases and are of high clinical relevance (Alcock et al. 2020; Avci et al. 2022). They are found on both integrons and plasmids (Brown & Amyes 2006). BLAST of the OXA annotation on a *Synechococcus* contig did suggest that it might be a class-D beta-lactamase, as there are multiple gene hits for *Synechococcus* class-D beta-lactamases. A previous paper did a comprehensive review of non-clinical bacteria encoding beta-lactamases and listed cyanobacteria as possessors of an OXA-domain family, including cyanobacteria that had class D beta-lactamase homologs on plasmids (Lupo et al. 2022).

**Fig. 7:**
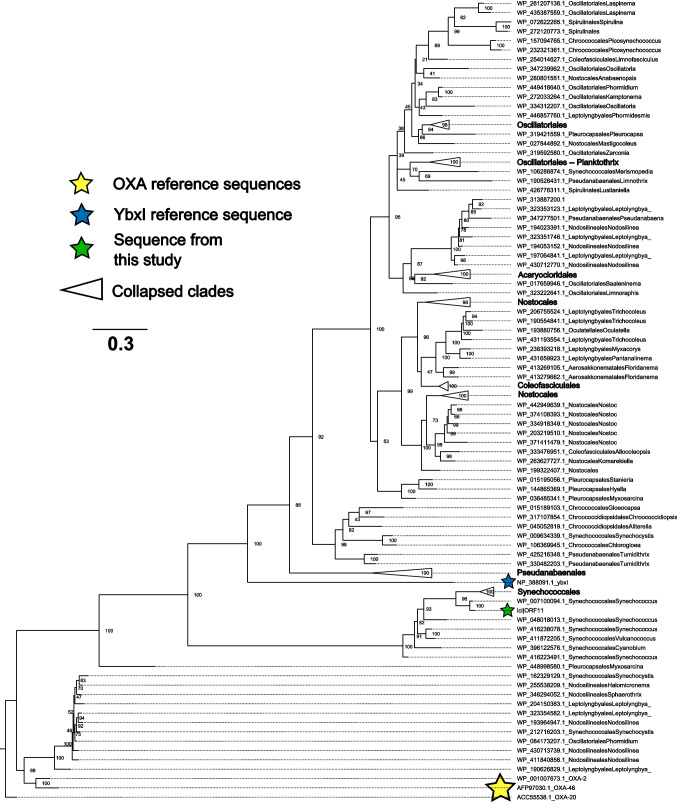
Maximum likelihood tree of cyanobacteria OXA/*ybxI* homologous amino acid sequences. OXA-20 representative sequence from *Acinetobacter baumannii* is shown at the root as the outgroup. Branch length indicates the expected number of substitutions per site. Node labels show ultrafast bootstrap support (% of bootstrap replicates). Clades composed of the same family were collapsed; in these cases, the family name is shown. Tip labels show NCBI RefSeq accession number, family, and genus (if taxonomic data was available)

The maximum likelihood tree provides further support for the intrinsic and conserved nature of the OXA-like homology in multiple families of cyanobacteria (Fig. 7). *YbxI* is an experimentally verified class-D beta-lactamase that has extremely low levels of beta-lactamase catalytic activity (Colombo et al. 2004). Most sequences clustered in a large clade with *YbxI*, suggesting they also might have extremely low (or negligible) class-D beta-lactamase activity. However, there is a smaller clade of cyanobacteria that contains the OXA-2 and OXA-46 reference sequences in the tree, suggesting there are some cyanobacteria that could have higher beta-lactamase activity relative to *YbxI*. OXA-2 and OXA-46 are generally considered to be narrow spectrum (Antunes et al. 2014; Poirel et al. 2010). A past phylogenetic study established a cluster with OXA-2, OXA-20, and OXA-46 and noted cyanobacteria as a phylum with homologous sequences to this cluster (among Alphaproteobacteria, Betaproteobacteria, and Gammaproteobacteria) (Lupo et al. 2022). We expand on this work here with a direct focus on cyanobacteria.

This study was not able to measure ARG transmission but was an exploratory look at ARGs and cyanobacteria in the freshwater metagenome. Overall, cyanobacteria were annotated as ARG hosts using both tools and have homologous genes to common ARG domains, but do not seem to be significant reservoirs of ARGs to drugs of last resort, at least in this dataset.

## Conclusions

Our primary objectives were to (1) identify the presence of cyanobacteria-hosted ARGs and (2) quantify cyanobacteria, ARGs, and cyanotoxins. We found that *Microcystis* dominated the cyanobacteria community in Lake Erie and *Planktothrix* dominated in GLSM, while GLSM samples had higher microcystin concentrations. In these samples, cyanobacteria seem most likely to be conferring putative intrinsic resistance to antibiotics. Even so, cyanobacteria as a potential reservoir of ARGs deserves further study as cyanoHABs already pose serious public health concerns because of cyanotoxins. This study is an example of using shotgun sequencing for detecting functional gene presence in metagenomic samples (Miłobedzka et al., 2022).

Future work with increased sample sizes would add to existing knowledge on cyanobacteria-hosted ARG abundances that may result in increased risks to human health. Major questions remain about the mobility of ARGs potentially hosted by cyanobacteria. To address this, future work should focus on ARG transmission in cyanobacteria and other bacteria, especially in water that contains pathogens, clinically relevant ARGs, and/or low levels of antibiotics that provide selection pressure (Fröhlich et al. 2021). The use of long-read sequencing could provide more confident ARG assignment to hosts at finer taxonomic levels (Portik et al., 2022). Future studies should also take advantage of data already available on public databases to perform ARG screening and homology searches from a cyanobacteria lens.

This study highlights that cyanotoxins remain present in drinking water sources. Advanced water treatments in place for cyanobacteria and cyanotoxins in these locations are known to generally be effective (Westrick et al., 2010). Treatment of AR in drinking water is less understood and not regulated. Research shows that advanced drinking water treatments such as biological activated carbon might encourage AR transmission between bacteria in biofilms (Duarte et al., 2022; Sanganyado & Gwenzi 2019; Tan et al., 2019), indicating cyanobacteria and cyanotoxin treatment processes are not necessarily effective for treating AR. This is especially important in a changing climate, where environmental contamination and cyanoHABs may increase (O’Neil et al. 2012) together with global warming. Overall, our study is novel for comparing multiple environmental contaminants in drinking water sources that may necessitate diverse water treatment considerations and historically are rarely considered in parallel.

## Supplementary Information

Below is the link to the electronic supplementary material.

ESM 1 (DOCX 1.60 MB)

## Data Availability

Sequencing data can be accessed using the accession number PRJNA1124395 on the National Center for Biotechnology Information (NCBI) Sequence Read Archive (SRA) (https://www.ncbi.nlm.nih.gov/sra/PRJNA1124395).

## Abbreviations

GLSM: Grand Lake St. Marys
cyanoHABs: Cyanobacterial harmful algal blooms
AR: Antibiotic resistance
ARGs: Antibiotic resistance genes
ddPCR: DropletDigital™ PCR
MAGs: Metagenome assembled genomes
