# Supplementary material for: Investigation of cyanobacteria-hosted antibiotic resistance genes in cyanoHAB-impacted drinking water sources
Source: Environ Sci Pollut Res Int. 2026 Mar 31;33(13):6140–64. doi: 10.1007/s11356-026-37644-9 (PMC13095955; doi:10.1007/s11356-026-37644-9)
Supplement: Supplementary file 1 — (DOCX 1.60 MB) [file 11356_2026_37644_MOESM1_ESM.docx]

**Supplementary Information**

**Investigation of Cyanobacteria-hosted Antibiotic Resistance Genes in cyanoHAB-Impacted Drinking Water Sources**

Abigail Volk^a,b^, Molly Mills^a^, Soryong Chae^c^, Jiyoung Lee^a,b,d,e*^

^a^Division of Environmental Health Sciences, College of Public Health, The Ohio State University, Columbus, OH, USA

^b^Environmental Sciences Graduate Program, The Ohio State University, Columbus, OH, USA

^c^Department of Chemical and Environmental Engineering, University of Cincinnati, Cincinnati, OH, USA

^d^Department of Food Science & Technology, The Ohio State University, Columbus, OH, USA

^e^Infectious Diseases Institute, The Ohio State University, Columbus, OH, USA

*Corresponding author: [lee.3598@osu.edu](mailto:lee.3598@osu.edu), Address: Cunz Hall, 1841 Neil Ave, Columbus, OH 43210, USA


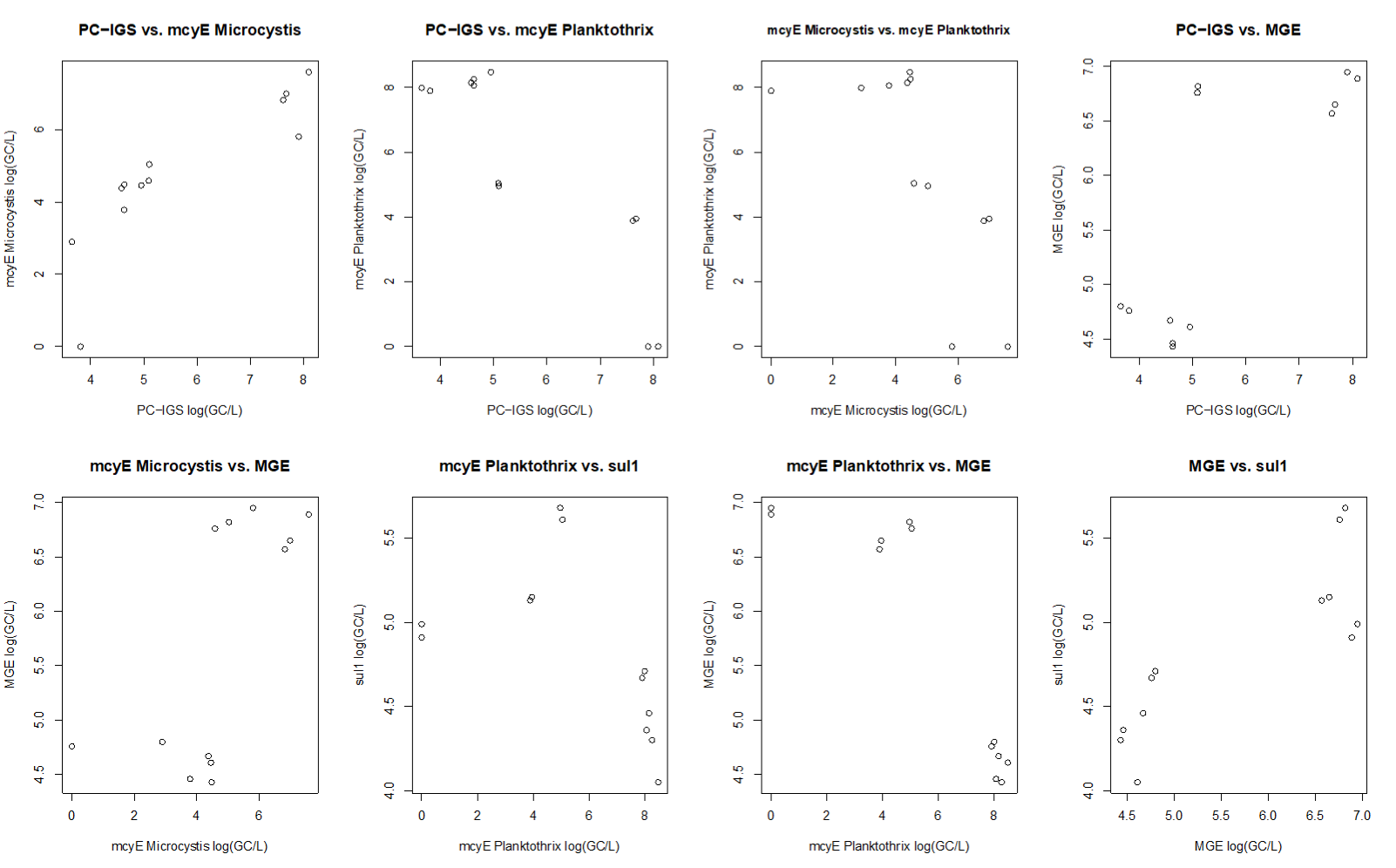


**Figure S1**. Scatterplots of correlations performed for each ddPCR gene combination.


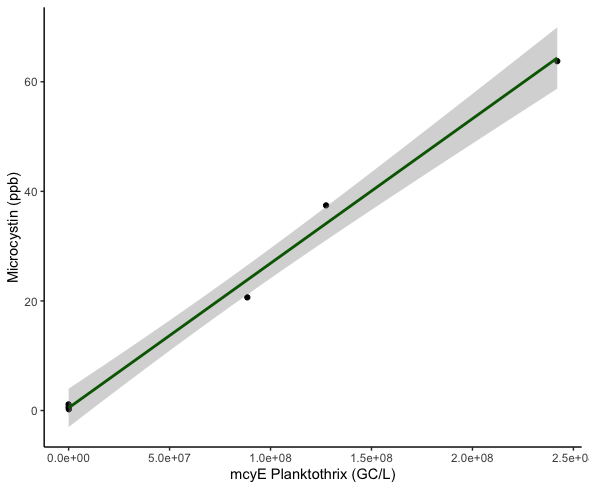


**Figure S2.** Ordinary least squares regression between microcystin concentration (ppb) and *Planktothrix mcyE* (GC/L). The results are shown in Supplementary Table 6.


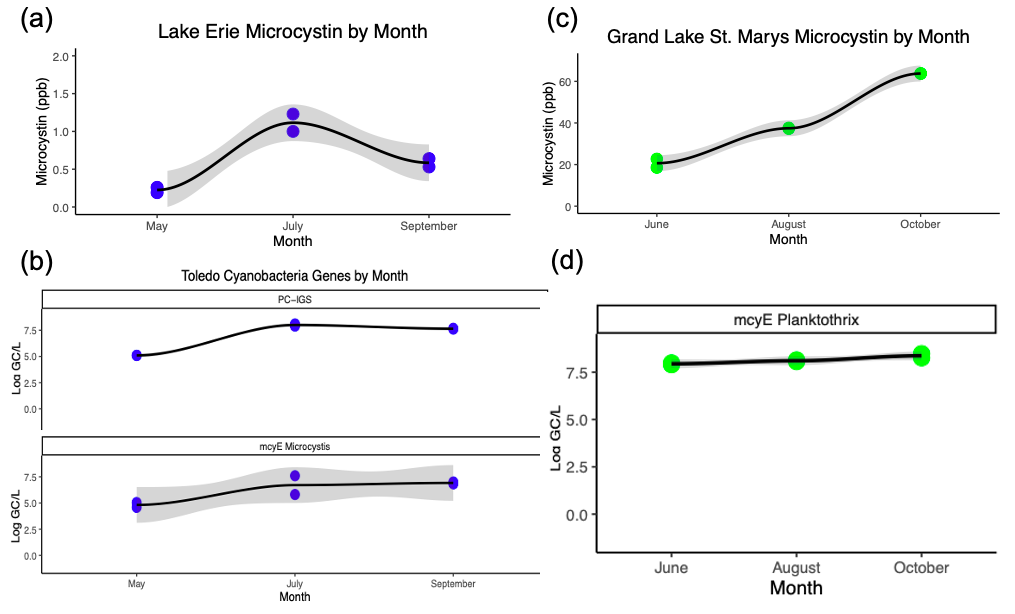


**Figure S3.** (a) Lake Erie microcystin (ppb) and (b) *Microcystis* ddPCR concentrations (PC-IGS and *mcyE Microcystis*, GC/L*)* from the May, June, and July measurements. (c) GLSM microcystin (ppb) and (d) *mcyE Planktothrix* ddPCR concentration (GC/L) from the June, August, and September measurements.


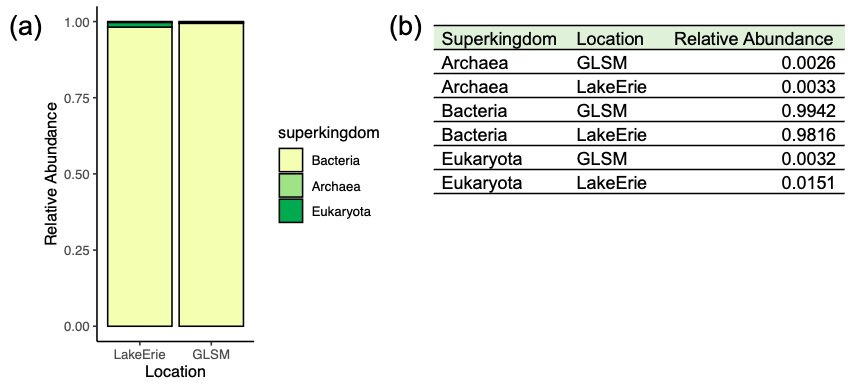


**Figure S4.** Superkingdom average relative abundance (a) barcharts and (b) percentages by location, calculated from CAT annotations.


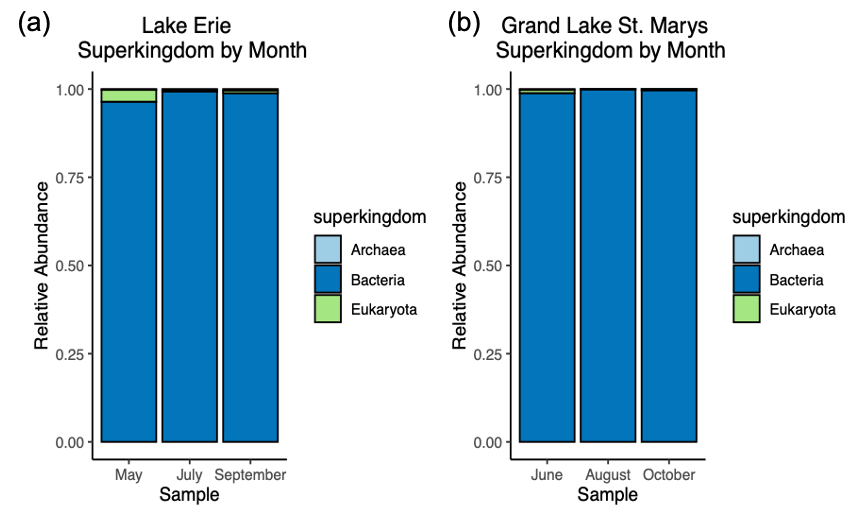


**Figure S5.** Superkingdom relative abundance by month for (a) Lake Erie and (b) Grand Lake St. Marys.


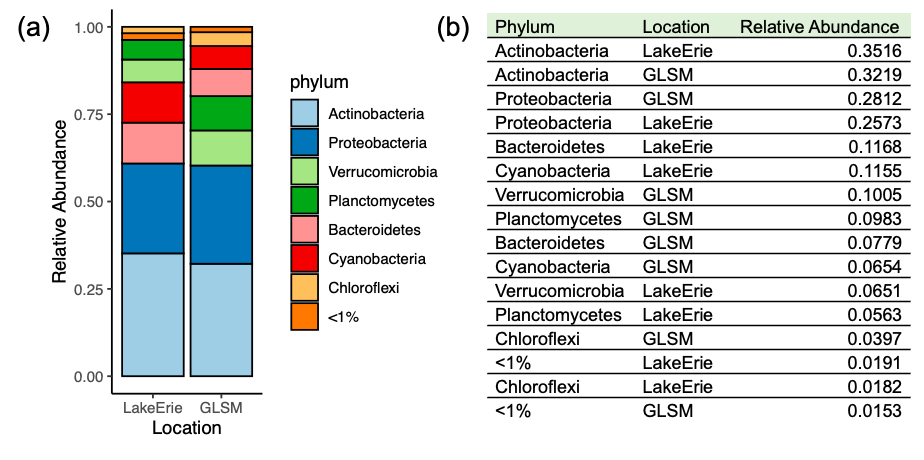


**Figure S6**. Bacteria phyla average relative abundance (a) barcharts and (b) percentages by location.


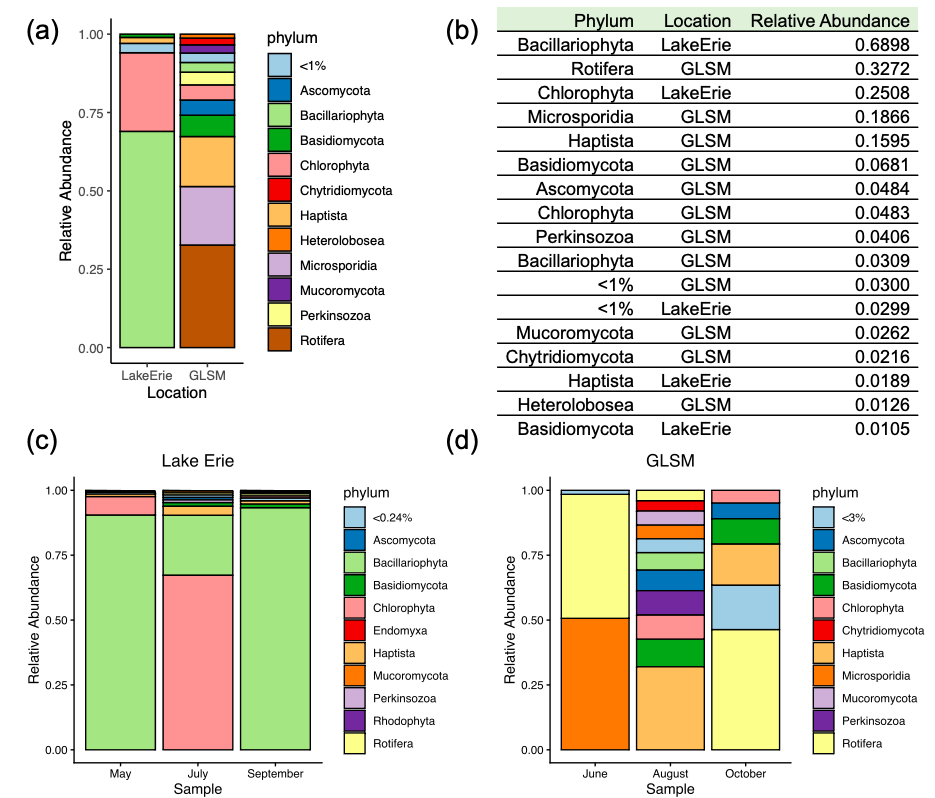


**Figure S7.** Eukaryotic phyla average relative abundance (a) average location barcharts, (b) percentages by location, (c) Lake Erie barcharts by month, and (d) GLSM barcharts by month.


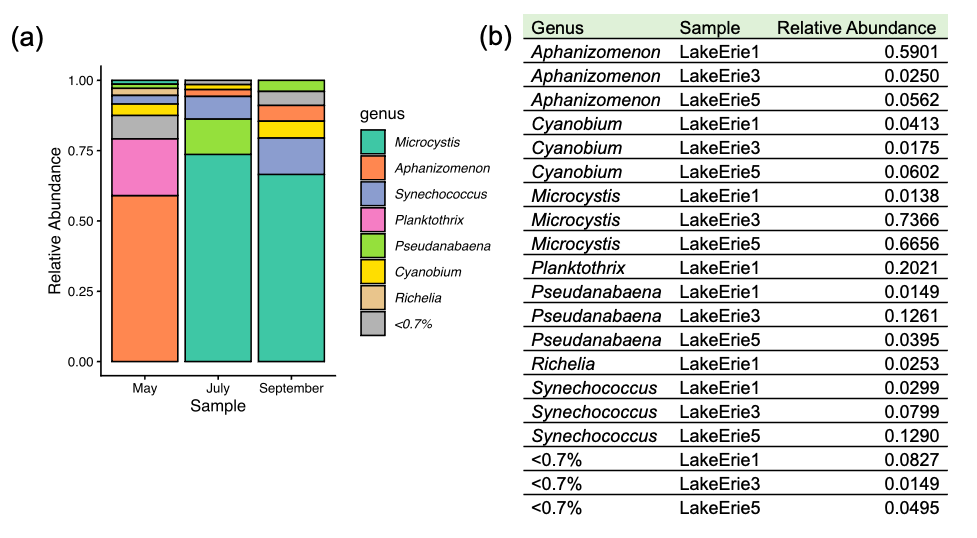


**Figure S8.** Lake Erie Cyanobacteria genera by month average relative abundance (a) barcharts and (b) percentages. LakeErie1 = May, LakeErie3 = July, LakeErie5 = September.


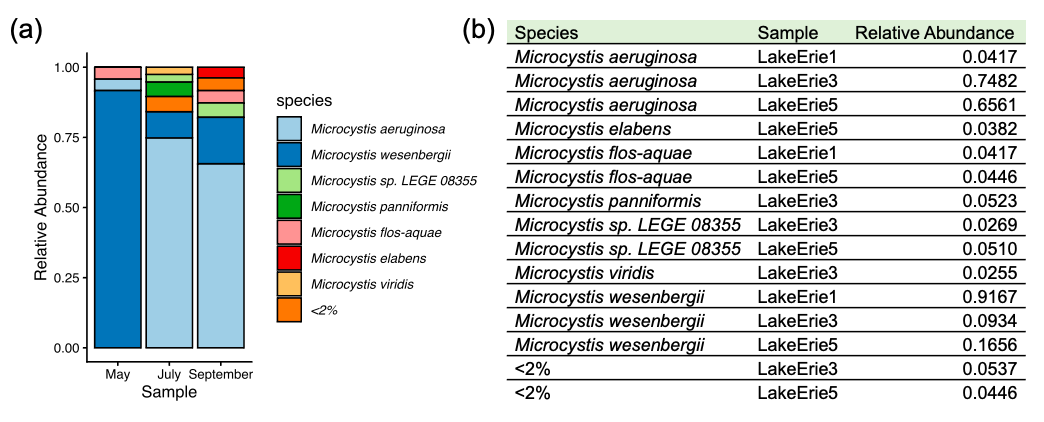


**Figure S9**. Lake Erie *Microcystis* spp. by month average relative abundance (a) barcharts and (b) percentages. LakeErie1 = May, LakeErie3 = July, LakeErie5 = September.


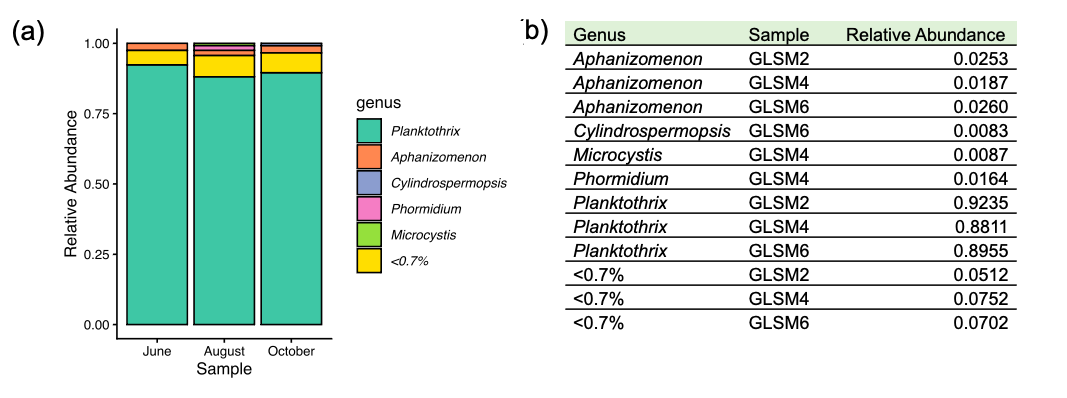


**Figure S10.** GLSM Cyanobacteria genera by month average relative abundance (a) barcharts and (b) percentages. GLSM2 = June, GLSM4 = August, GLSM6 = October.

**
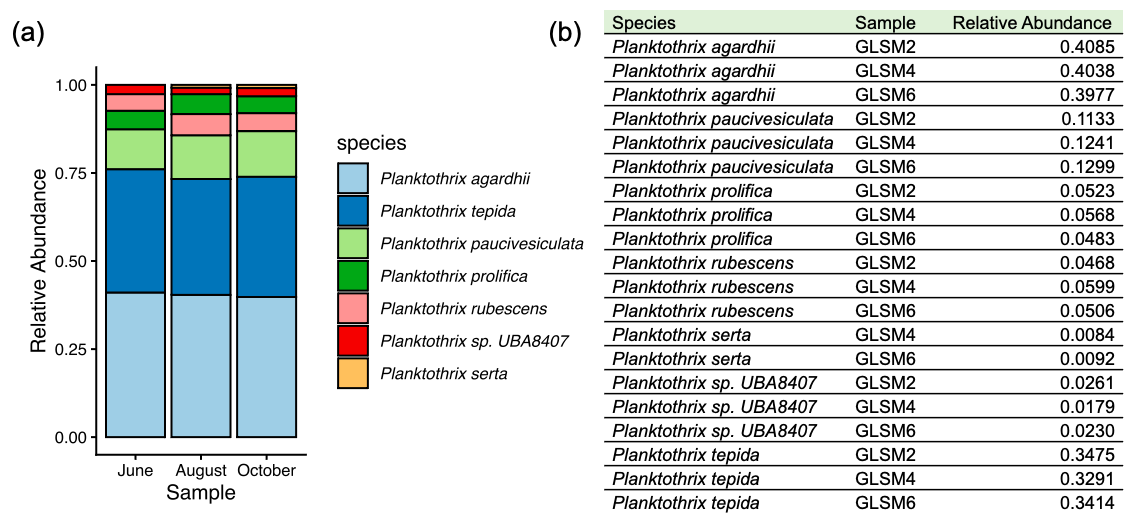
Figure S11.** GLSM *Planktothrix spp.* by month average relative abundance (a) barcharts and (b) percentages. GLSM2 = June, GLSM4 = August, GLSM6 = October.


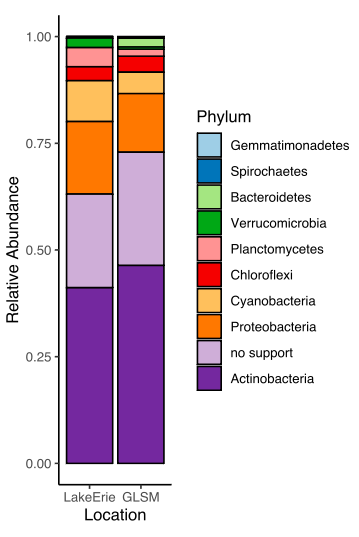


**Figure S12.** Average ARG host relative abundance. Data combined from both RGI and DeepARG.


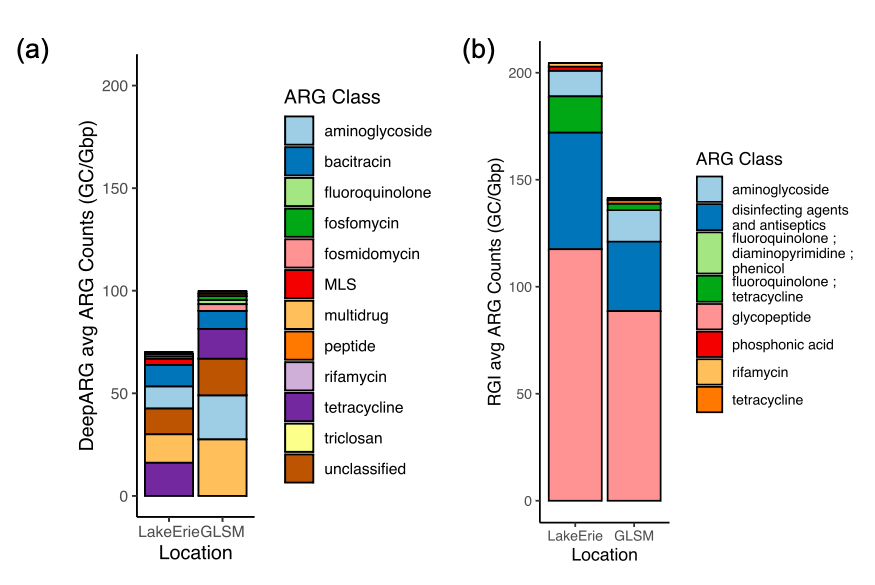


**Figure S13.** Average ARG class abundance by month annotated by (a) DeepARG and (b) RGI.


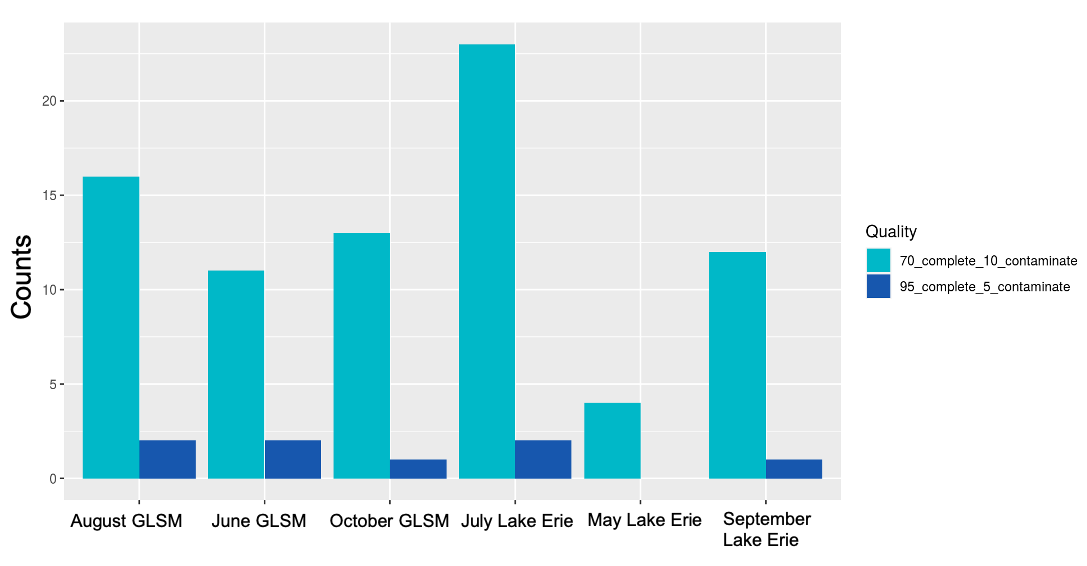
 **Figure S14.** The number of quality bins for each sequencing sample. MAGs with >70% completion and <10% are medium quality, and MAGs with >95% completion and <5% contamination are high quality.


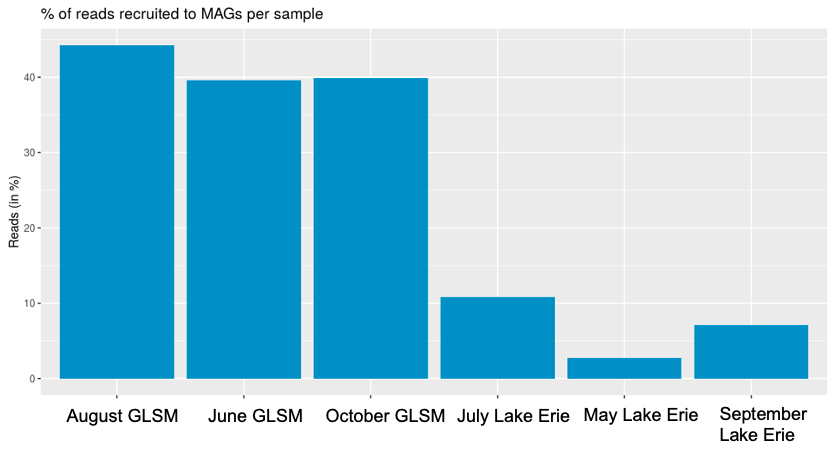


**Figure S15.** The number of reads recruited to MAGs for each sequencing sample.

**Table S1**. Sample information table. The number of samples processed for each assay is included for each site and respective visit date.

| Visit Date | Site | Metagenomic sequencing samples (#) | ddPCR samples (# technical replicates) | Cyanotoxin samples (# technical replicates) |
| --- | --- | --- | --- | --- |
| May 15^th^, 2022 | Lake Erie | 1 | 2 | 2 |
| June 27^th^, 2022 | GLSM | 1 | 2 | 2 |
| July 19^th^, 2022 | Lake Erie | 1 | 2 | 2 |
| August 16^th^, 2022 | GLSM | 1 | 2 | 2 |
| September 16^th^, 2022 | Lake Erie | 1 | 2 | 2 |
| October 14^th^, 2022 | GLSM | 1 | 2 | 2 |
| **Totals** |  | **6** | **12** | **12** |

**Table S2**. ddPCR gene detection and quantification conditions and primer information.

| Gene | Reaction Mixture | Thermal Cycler Conditions | Primer Sequences | Source |
| --- | --- | --- | --- | --- |
| *Microcystis* PC-IGS | Probe-based Bio-Rad supermix, 10 µL  1 uL primer and probe (FAM) µL  + PCR-grade water and template to 20 µL | 1. 95 10min 2. 94 30s 3. 59 30s 4. 60 30s 5. Return to 2 37x 6. 98 10min | F: GCTACTTCGACCGCGCC  R: TCCTACGGTTTAATTGAGACTAGCC  Probe: CCGCTGCTGTCGCCTAGTCCCTG | (Kurmayer & Kutzenberger, 2003) |
| *mcyE Microcystis* | Probe-based Bio-Rad supermix, 10 µL  1 uL primer and probe (FAM) µL  + PCR-grade water and template to 20 µL | 1. 95 10min 2. 94 30s 3. 56 30s 4. 60 30s 5. Return to 2 37x 6. 98 10min | F: CAATGGGAGCATAACGAGTCAA  R: AAGCAAACTGCTCCCGGTATC  Probe: CAATGGTTATCGAATTGACCCCGGAGAAAT | (Sipari et al., 2010) |
| *mcyE Planktothrix* | Probe-based Bio-Rad supermix, 10 µL  1 uL primer and probe (FAM) µL  + PCR-grade water and template to 20 µL | 1. 95 10min 2. 94 30s 3. 59 30s 4. 60 30s 5. Return to 2 37x 6. 98 10min | F: GATTGCACTCAATGAAACCG  R: AACGTGGGTTACGATTCTCG  FAM- TTGGCGGACATTCTCTGATGCTTT | (Ngwa et al., 2014) |
| *anaC* | Evagreen Supermix 10 µL  Biorad Primers 1 µL  + PCR-grade water and template to 20 µL | 1. 95 5min 2. 95 30s 3. 57 30s 4. 60 30s 5. Return to 2 37x 6. 4 5min 7. 90 5min | F: TCTGGTATTCAGTCCCCTCTAT  R: CCCAATAGCCTGTCATCAA | (Rantala-Ylinen et al., 2011) |
| *sxtA* | Evagreen Supermix 10 µL  sxtAF 1 µL  sxtAR 1 µL  + PCR-grade water and template to 20 µL | 1. 95 5min 2. 95 30s 3. 57 30s 4. 60 30s 5. Return to 2 37x 6. 4 5min 7. 90 5min | F: CTGAGCAAGGCGTTCAATTC  R: TACAGATMGGCCCTGTGARC | (S. A. Murray et al., 2011) |
| *intI1* (MGE) | Probe superm 10 µL  1 uL primer and probe (FAM) µL  + PCR-grade water and template to 20 µL | 1. 95 10min 2. 94 30s 3. 59 30s 4. 60 30s 5. Return to 2 40x 6. 98 10min | F: GCCTTGATGTTACCCGAGAG  R: GATCGGTCGAATGCGTGT  P: [FAM]-ATTCCTGGCCGTGGTTCTGGGTTTT–[BHQ] | (González-Plaza et al., 2019) |
| *sul1* | Evagreen Supermix 10 µL  sul1 1 µL  + PCR-grade water and template to 20 µL | 1. 95 5min 2. 95 30s 3. 60 1 min 4. Return to 2 44x 5. 4 5min 6. 90 5min | F: CACCGGAAACATCGCTGCA  R: AAGTTCCGCCGCAAGGCT | (Luo et al., 2010) |
| *tetQ* | Evagreen Supermix 10 µL  tetQ 1 µL  + PCR-grade water and template to 20 | 1. 95 5min 2. 95 30s 3. 60 1 min 4. Return to 2 44x 5. 4 5min 6. 90 5min | F: CATGGATCAGCAATGTTCAATATCGG  R: CCTGGATCCACAATGTATTCAGAGCGG | (Luo et al., 2010) |

**Table S3**. Sequencing information: read numbers, assembly information, and alpha diversity for each sequencing sample.

| Sample | Raw Reads (#) | Cleaned Reads (#) | Assembly Reads (#) | Assembly sum (bp) | Assembly max (bp) | Average contig length (bp) | Microbial Community Alpha Diversity (Shannon index) |
| --- | --- | --- | --- | --- | --- | --- | --- |
| Toledo May | 62631788 | 62615395 | 1052937 | 581694198 | 105726 | 552.4 | 3.72 |
| Celina June | 77886631 | 77870349 | 441718 | 294294335 | 135368 | 666.2 | 3.82 |
| Toledo July | 88180523 | 88160711 | 1163470 | 734413841 | 110184 | 631.2 | 3.81 |
| Celina August | 86148706 | 86132997 | 606354 | 397667923 | 133154 | 655.8 | 3.76 |
| Toledo September | 49496897 | 49482332 | 945931 | 598439609 | 175329 | 632.6 | 3.83 |
| Celina October | 47041386 | 47029164 | 490071 | 317019694 | 238123 | 646.9 | 4.00 |

**Table S4**. Phyla included in community analysis for each superkingdom.

| Superkingdom | Bacteria | Archaea | Eukaryota |
| --- | --- | --- | --- |
| Phyla | All | All | Ascomycota, Bacillariophyta, Basidiomycota, Blastocladiomycota, Chloropyta, Chytridiomycota, Ciliophora, Cryptomycota, Discosea, Endomyxa, Euglenazoa, Evosea, Foraminifera, Haptista, Heterolobosea, Imbricatea, Microsopridia, Mucuromycota, Parabasalia, Perkinsozoa, Placozoa, Preaxostyla, Rhodophyta, Rotifera, Tardigrada, Zoopagomycota |

**Table S5**. PCA loadings.

|  | PC1 | PC2 | PC3 |
| --- | --- | --- | --- |
| *PC-IGS* | 0.368 | -0.411 | -0.099 |
| *mcyE Microcystis* | 0.367 | -0.414 | -0.110 |
| *mcyE Planktothrix* | -0.460 | -0.103 | -0.125 |
| *sul1* | 0.265 | 0.508 | -0.077 |
| *tetQ* | 0.042 | 0.406 | -0.570 |
| *MGE* | 0.465 | -0.019 | -0.199 |
| Microcystin | -0.456 | -0.099 | -0.180 |
| *sxtA* | 0.148 | 0.464 | 0.367 |
| *anaC* | -0.030 | 0.030 | -0.652 |

**Table S6.** Results of linear regression between microcystin concentration (ppb) and *Planktothrix mcyE* (GC/L).

|  | Estimate | Std. Error | t value | Pr(>\|t\|) |  |
| --- | --- | --- | --- | --- | --- |
| (Intercept) | 4.823e-01 | 1.259 | 0.383 | 0.721 |  |
| Avg *Planktothrix mcyE* | 2.639e-07 | 1.072e-08 | 24.610 | 1.62e-05 | *** |

**Table S6**. Hits in which the same or similar genes were annotated using both tools. GLSM2 = June, GLSM4 = August, GLSM6 = October. LakeErie1 = May, LakeErie3 = July, LakeErie5 = September.


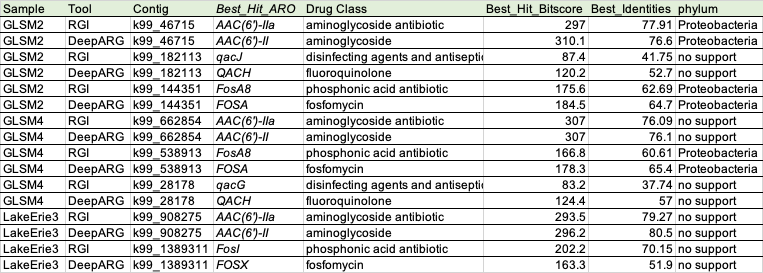


**Table S7**. Hits in which the same gene was annotated differently between tools. GLSM2 = June, GLSM4 = August, GLSM6 = October. LakeErie1 = May, LakeErie3 = July, LakeErie5 = September.

| Sample | Tool | Contig | ARG | ARG Class | Bitscore | Identity | phylum |
| --- | --- | --- | --- | --- | --- | --- | --- |
| GLSM2 | RGI | k99_352692 | *adeF* | fluoroquinolone antibiotic; tetracycline antibiotic | 805.4 | 43.12 | no support |
| GLSM2 | RGI | k99_352692 | *adeF* | fluoroquinolone antibiotic; tetracycline antibiotic | 791.2 | 41.64 | no support |
| GLSM2 | DeepARG | k99_352692 | *MEXF* | multidrug | 1174.8 | 58.8 | no support |
| GLSM2 | RGI | k99_296141 | *adeF* | fluoroquinolone antibiotic; tetracycline antibiotic | 1062 | 54.89 | Proteobacteria |
| GLSM2 | DeepARG | k99_296141 | *MEXF* | multidrug | 1133.6 | 57.9 | Proteobacteria |
| GLSM4 | RGI | k99_88129 | *qacJ* | disinfecting agents and antiseptics | 82.4 | 41.18 | Proteobacteria |
| GLSM4 | DeepARG | k99_88129 | *PSEUDOMONAS_AERUGINOSA_EMRE* | aminoglycoside | 112.5 | 52.5 | Proteobacteria |
| GLSM4 | RGI | k99_71845 | *qacJ* | disinfecting agents and antiseptics | 84.7 | 39.25 | Proteobacteria |
| GLSM4 | DeepARG | k99_71845 | *PSEUDOMONAS_AERUGINOSA_EMRE* | aminoglycoside | 124.4 | 59 | Proteobacteria |
| GLSM4 | RGI | k99_592159 | *Mycobacterium tuberculosis rpsL mutations conferring resistance to Streptomycin* | aminoglycoside antibiotic | 208 | 84.55 | Actinobacteria |
| GLSM4 | DeepARG | k99_592159 | *RPOB2* | multidrug | 1263.8 | 56.2 | Actinobacteria |
| GLSM4 | RGI | k99_527737 | *qacJ* | disinfecting agents and antiseptics | 97.1 | 42.06 | Proteobacteria |
| GLSM4 | DeepARG | k99_527737 | *PSEUDOMONAS_AERUGINOSA_EMRE* | aminoglycoside | 122.1 | 56.2 | Proteobacteria |
| GLSM4 | RGI | k99_523040 | *qacJ* | disinfecting agents and antiseptics | 84 | 40.57 | no support |
| GLSM4 | DeepARG | k99_523040 | *PSEUDOMONAS_AERUGINOSA_EMRE* | aminoglycoside | 118.6 | 56.6 | no support |
| GLSM4 | RGI | k99_520284 | *adeF* | fluoroquinolone antibiotic; tetracycline antibiotic | 786.2 | 43.05 | Proteobacteria |
| GLSM4 | DeepARG | k99_520284 | *MEXF* | multidrug | 1087.8 | 55.2 | Proteobacteria |
| GLSM4 | RGI | k99_279315 | *qacG* | disinfecting agents and antiseptics | 86.3 | 42.72 | Proteobacteria |
| GLSM4 | DeepARG | k99_279315 | *ABES* | multidrug | 120.9 | 59.6 | Proteobacteria |
| GLSM4 | RGI | k99_184886 | *adeF* | fluoroquinolone antibiotic; tetracycline antibiotic | 889.4 | 47.72 | Planctomycetes |
| GLSM4 | DeepARG | k99_184886 | *MEXF* | multidrug | 962.6 | 51.3 | Planctomycetes |
| GLSM4 | RGI | k99_140422 | *adeF* | fluoroquinolone antibiotic; tetracycline antibiotic | 817.8 | 47.09 | Planctomycetes |
| GLSM4 | DeepARG | k99_140422 | *MEXF* | multidrug | 886.3 | 50.1 | Planctomycetes |
| GLSM6 | RGI | k99_533646 | *qacG* | disinfecting agents and antiseptics | 86.3 | 42.72 | Proteobacteria |
| GLSM6 | DeepARG | k99_533646 | *ABES* | multidrug | 120.9 | 59.6 | Proteobacteria |
| GLSM6 | RGI | k99_471385 | *adeF* | fluoroquinolone antibiotic; tetracycline antibiotic | 788.5 | 42.95 | Proteobacteria |
| GLSM6 | DeepARG | k99_471385 | *MEXF* | multidrug | 1089.3 | 55.2 | Proteobacteria |
| GLSM6 | RGI | k99_381701 | *qacJ* | disinfecting agents and antiseptics | 83.2 | 40.86 | Proteobacteria |
| GLSM6 | DeepARG | k99_381701 | *PSEUDOMONAS_AERUGINOSA_EMRE* | aminoglycoside | 114.4 | 59.1 | Proteobacteria |
| GLSM6 | RGI | k99_263269 | *qacG* | disinfecting agents and antiseptics | 83.2 | 36.79 | no support |
| GLSM6 | DeepARG | k99_263269 | *PSEUDOMONAS_AERUGINOSA_EMRE* | aminoglycoside | 111.3 | 58.1 | no support |
| LakeErie3 | RGI | k99_900067 | *adeF* | fluoroquinolone antibiotic; tetracycline antibiotic | 878.6 | 47.64 | Planctomycetes |
| LakeErie3 | DeepARG | k99_900067 | *MEXF* | multidrug | 957.6 | 51.2 | Planctomycetes |
| LakeErie3 | RGI | k99_788235 | *qacG* | disinfecting agents and antiseptics | 77.4 | 36.54 | Proteobacteria |
| LakeErie3 | DeepARG | k99_788235 | *EMRE* | multidrug | 114.4 | 55.8 | Proteobacteria |
| LakeErie3 | RGI | k99_683067 | *qacG* | disinfecting agents and antiseptics | 92 | 43.4 | no support |
| LakeErie3 | DeepARG | k99_683067 | *PSEUDOMONAS_AERUGINOSA_EMRE* | aminoglycoside | 117.5 | 52.8 | no support |
| LakeErie3 | RGI | k99_339028 | *qacG* | disinfecting agents and antiseptics | 92.8 | 43 | Actinobacteria |
| LakeErie3 | DeepARG | k99_339028 | *EMRE* | multidrug | 94.7 | 51.1 | Actinobacteria |
| LakeErie3 | RGI | k99_1128338 | *qacG* | disinfecting agents and antiseptics | 100.9 | 44.34 | Planctomycetes |
| LakeErie3 | DeepARG | k99_1128338 | *PSEUDOMONAS_AERUGINOSA_EMRE* | aminoglycoside | 130.6 | 57.4 | Planctomycetes |
| LakeErie3 | RGI | k99_1048445 | *qacG* | disinfecting agents and antiseptics | 92 | 44.34 | no support |
| LakeErie3 | DeepARG | k99_1048445 | *PSEUDOMONAS_AERUGINOSA_EMRE* | aminoglycoside | 119.4 | 53.8 | no support |
| LakeErie5 | RGI | k99_63389 | *adeF* | fluoroquinolone antibiotic; tetracycline antibiotic | 949.9 | 48.95 | no support |
| LakeErie5 | DeepARG | k99_63389 | *MEXF* | multidrug | 1033.9 | 52.7 | no support |
| LakeErie5 | RGI | k99_598376 | *qacG* | disinfecting agents and antiseptics | 93.6 | 43.4 | no support |
| LakeErie5 | DeepARG | k99_598376 | *PSEUDOMONAS_AERUGINOSA_EMRE* | aminoglycoside | 118.2 | 53.8 | no support |
| LakeErie5 | RGI | k99_110414 | *Mycobacterium tuberculosis rpsL mutations conferring resistance to Streptomycin* | aminoglycoside antibiotic | 206.8 | 86.07 | Actinobacteria |
| LakeErie5 | DeepARG | k99_110414 | *RPOB2* | multidrug | 1318.5 | 58.8 | Actinobacteria |

**Table S8**. ARG Host Average Relative Abundance by Location. DeepARG and RGI results were merged and checked for duplicate contig annotations before computing relative abundance of hosts.

| Phylum | Location | Relative Abundance |
| --- | --- | --- |
| Actinobacteria | GLSM | 0.4640 |
| Actinobacteria | LakeErie | 0.4120 |
| no support | GLSM | 0.2650 |
| no support | LakeErie | 0.2190 |
| Proteobacteria | LakeErie | 0.1698 |
| Proteobacteria | GLSM | 0.1373 |
| Cyanobacteria | LakeErie | 0.0957 |
| Cyanobacteria | GLSM | 0.0502 |
| Planctomycetes | LakeErie | 0.0449 |
| Chloroflexi | GLSM | 0.0376 |
| Chloroflexi | LakeErie | 0.0330 |
| Verrucomicrobia | LakeErie | 0.0221 |
| Bacteroidetes | GLSM | 0.0212 |
| Planctomycetes | GLSM | 0.0164 |
| Verrucomicrobia | GLSM | 0.0050 |
| Spirochaetes | LakeErie | 0.0035 |
| Gemmatimonadetes | GLSM | 0.0032 |
| Bacteroidetes | LakeErie | 0.0000 |
| Gemmatimonadetes | LakeErie | 0.0000 |
| Spirochaetes | GLSM | 0.0000 |

**Table S9**. DeepARG “possible” hits (probability <80%) annotated to Cyanobacterial contigs. OXA and *Pseudomonas aeruginosa* *emrE* are unique ARGs that were not annotated in the high confidence table. GLSM2 = June, GLSM4 = August, GLSM6 = October. LakeErie1 = May, LakeErie3 = July, LakeErie5 = September.

**Table S10**. Cyanobacteria MAG taxonomy, completeness, and contamination. S = sample number.

| Bin | Taxonomy | Completeness | Contamination |
| --- | --- | --- | --- |
| S2.bin2 | *d__Bacteria;p__Cyanobacteria;c__Cyanobacteriia;o__Cyanobacteriales;f__Microcoleaceae;g__Planktothrix;s__Planktothrix agardhii* | 94.10 | 0.655 |
| S3.bin3 | *d__Bacteria;p__Cyanobacteria;c__Cyanobacteriia;o__PCC-6307;f__Cyanobiaceae;g__Cyanobium;s__* | 91.98 | 1.630 |
| S5.bin7 | *d__Bacteria;p__Cyanobacteria;c__Cyanobacteriia;o__Cyanobacteriales;f__Microcystaceae;g__Microcystis;s__Microcystis aeruginosa* | 81.42 | 4.705 |

**Table S11.** Taxonomy and RGI ARG Hits for all MAGs. All hits are classified as strict.

| Bin name(s) | *Taxonomy classification* | # ARGs annotated | ARO Term | Detection Criteria | Drug Class | Resistance Mechanism | Identity of Matching Region (%) | Length of Reference Sequence (%) |
| --- | --- | --- | --- | --- | --- | --- | --- | --- |
| S1.bin1 | *d__Bacteria;p__Bacteroidota;c__Bacteroidia;o__Flavobacteriales;f__Schleiferiaceae;g__TMED14;s__* | None |  |  |  |  |  |  |
| S1.bin2 | *d__Bacteria;p__Proteobacteria;c__Gammaproteobacteria;o__Burkholderiales;f__Methylophilaceae;g__Methylopumilus;s__Methylopumilus planktonicus* | None |  |  |  |  |  |  |
| S1.bin3 | *d__Bacteria;p__Proteobacteria;c__Gammaproteobacteria;o__Burkholderiales;f__Burkholderiaceae;g__Polynucleobacter;s__Polynucleobacter sp002292975* | None |  |  |  |  |  |  |
| S1.bin4 | *d__Bacteria;p__Verrucomicrobiota;c__Verrucomicrobiae;o__Opitutales;f__UBA953;g__UBA953;s__UBA953 sp003569245* | 2 | *vanT gene in vanG cluster* | protein homolog model | glycopeptide | antibiotic target alteration | 34.49 | 53.51 |
|  |  |  | *adeF* | protein homolog model | fluoroquinolone, tetracycline | antibiotic efflux | 42.19 | 98.39 |
| S2.bin1 | *d__Bacteria;p__Proteobacteria;c__Gammaproteobacteria;o__Burkholderiales;f__Methylophilaceae;g__Methylopumilus;s__Methylopumilus rimovensis* | 1 | *vanT gene in vanG cluster* | protein homolog model | glycopeptide | antibiotic target alteration | 32.24 | 50.56 |
| S2.bin2 | *d__Bacteria;p__Cyanobacteria;c__Cyanobacteriia;o__Cyanobacteriales;f__Microcoleaceae;g__Planktothrix;s__Planktothrix agardhii* | 4 | *vanH gene in vanB cluster* | protein homolog model | glycopeptide | antibiotic target alteration | 39.93 | 103.41 |
|  |  |  | *qacJ* | protein homolog model | disinfecting agents and antiseptics | antibiotic efflux | 39.8 | 100 |
|  |  |  | *vanY gene in vanM cluster* | protein homolog model | glycopeptide | antibiotic target alteration | 34.35 | 124.46 |
|  |  |  | *vanT gene in vanG cluster* | protein homolog model | glycopeptide | antibiotic target alteration | 34.76 | 56.32 |
| S2.bin3 | *d__Bacteria;p__Actinobacteriota;c__Acidimicrobiia;o__Acidimicrobiales;f__Ilumatobacteraceae;g__UBA2093;s__* | 2 | *vanT gene in vanG cluster* | protein homolog model | glycopeptide | antibiotic target alteration | 35.42 | 52.81 |
|  |  |  | *Mycobacterium tuberculosis rpsL mutations conferring resistance to Streptomycin* | protein variant model | aminoglycoside | antibiotic target alteration | 86.18 | 99.19 |
| S2.bin4 | *d__Bacteria;p__Proteobacteria;c__Alphaproteobacteria;o__Pelagibacterales;f__Pelagibacteraceae;g__Fonsibacter;s__Fonsibacter sp018882565* | None |  |  |  |  |  |  |
| S2.bin5 | *d__Bacteria;p__Proteobacteria;c__Gammaproteobacteria;o__Burkholderiales;f__Burkholderiaceae;g__RFTU01;s__* | None |  |  |  |  |  |  |
| S2.bin6 | *d__Bacteria;p__Proteobacteria;c__Gammaproteobacteria;o__Methylococcales;f__Methylococcaceae;g__UBA6136;s__* | 3 | *adeF* | protein homolog model | fluoroquinolone, tetracycline | antibiotic efflux | 42.26 | 98.96 |
|  |  |  | *vanT gene in vanG cluster* | protein homolog model | glycopeptide | antibiotic target alteration | 31.35 | 51.26 |
|  |  |  | *adeF* | protein homolog model | fluoroquinolone, tetracycline | antibiotic efflux | 54.89 | 99.43 |
| S2.bin7 | *d__Bacteria;p__Proteobacteria;c__Gammaproteobacteria;o__Burkholderiales;f__Burkholderiaceae;g__Polynucleobacter;s__* | None |  |  |  |  |  |  |
| S2.bin8 | *d__Bacteria;p__Verrucomicrobiota;c__Verrucomicrobiae;o__Chthoniobacterales;f__UBA6821;g__UBA6821;s__* | 5 | *adeF* | protein homolog model | fluoroquinolone, tetracycline | antibiotic efflux | 41.59 | 98.96 |
|  |  |  | *adeF* | protein homolog model | fluoroquinolone, tetracycline | antibiotic efflux | 43.12 | 98.11 |
|  |  |  | *adeF* | protein homolog model | fluoroquinolone, tetracycline | antibiotic efflux | 41.64 | 99.43 |
|  |  |  | *vanT gene in vanG cluster* | protein homolog model | glycopeptide | antibiotic target alteration | 37.29 | 52.95 |
|  |  |  | *qacJ* | protein homolog model | disinfecting agents and antiseptics | antibiotic efflux | 40.59 | 102.8 |
| S2.bin9 | *d__Bacteria;p__Actinobacteriota;c__Actinomycetia;o__Nanopelagicales;f__Nanopelagicaceae;g__Nanopelagicus;s__* | 1 | *vanY gene in vanF cluster* | protein homolog model | glycopeptide | antibiotic target alteration | 32.59 | 143.69 |
| S2.bin10 | *d__Bacteria;p__Actinobacteriota;c__Acidimicrobiia;o__Acidimicrobiales;f__Ilumatobacteraceae;g__UBA2093;s__* | 1 | *Mycobacterium tuberculosis rpsL mutations conferring resistance to Streptomycin* | protein variant model | aminoglycoside | antibiotic target alteration | 86.18 | 99.19 |
| S2.bin11 | *d__Bacteria;p__Actinobacteriota;c__Acidimicrobiia;o__Acidimicrobiales;f__Ilumatobacteraceae;g__F1-20-MAGs119;s__* | 1 | *Mycobacterium tuberculosis rpsL mutations conferring resistance to Streptomycin* | protein variant model | aminoglycoside | antibiotic target alteration | 85.37 | 99.19 |
| S3.bin1 | *d__Bacteria;p__Proteobacteria;c__Gammaproteobacteria;o__Burkholderiales;f__Burkholderiaceae;g__UBA954;s__UBA954 sp002293155* | 1 | *tetB(P)* | protein homolog model | tetracycline | antibiotic target protection | 40.1 | 96.17 |
| S3.bin2 | *d__Bacteria;p__Proteobacteria;c__Gammaproteobacteria;o__Xanthomonadales;f__Xanthomonadaceae;g__Arenimonas;s__* | None |  |  |  |  |  |  |
| S3.bin3 | *d__Bacteria;p__Cyanobacteria;c__Cyanobacteriia;o__PCC-6307;f__Cyanobiaceae;g__Cyanobium;s__* | None |  |  |  |  |  |  |
| S3.bin4 | *d__Bacteria;p__Actinobacteriota;c__Acidimicrobiia;o__Acidimicrobiales;f__Ilumatobacteraceae;g__F1-60-MAGs027;s__F1-60-MAGs027 sp017853375* | 1 | *Mycobacterium tuberculosis rpsL mutations conferring resistance to Streptomycin* | protein variant model | aminoglycoside | antibiotic target alteration | 84.55 | 99.19 |
| S3.bin5 | *d__Bacteria;p__Proteobacteria;c__Alphaproteobacteria;o__Acetobacterales;f__Acetobacteraceae;g__Roseomonas_B;s__Roseomonas_B sp903844785* | None |  |  |  |  |  |  |
| S3.bin6 | *d__Bacteria;p__Actinobacteriota;c__Acidimicrobiia;o__Acidimicrobiales;f__Ilumatobacteraceae;g__Casp-actino8;s__* | 2 | *vanT gene in vanG cluster* | protein homolog model | glycopeptide | antibiotic target alteration | 33.51 | 53.51 |
|  |  |  | *Mycobacterium tuberculosis rpsL mutations conferring resistance to Streptomycin* | protein variant model | aminoglycoside | antibiotic target alteration | 85.37 | 99.19 |
| S3.bin7 | *d__Bacteria;p__Bacteroidota;c__Bacteroidia;o__NS11-12g;f__UBA955;g__UBA955;s__UBA955 sp002293105* | None |  |  |  |  |  |  |
| S3.bin8 | *d__Bacteria;p__Bacteroidota;c__Bacteroidia;o__UBA7662;f__UBA7662;g__SYHX01;s__SYHX01 sp903856555* | None |  |  |  |  |  |  |
| S3.bin9 | *d__Bacteria;p__Proteobacteria;c__Gammaproteobacteria;o__Steroidobacterales;f__Steroidobacteraceae;g__UBA964;s__UBA964 sp903910215* | None |  |  |  |  |  |  |
| S3.bin10 | *d__Bacteria;p__Actinobacteriota;c__Acidimicrobiia;o__Acidimicrobiales;f__Ilumatobacteraceae;g__BACL27;s__BACL27 sp014190055* | None |  |  |  |  |  |  |
| S3.bin11 | *d__Bacteria;p__Actinobacteriota;c__Actinomycetia;o__Actinomycetales;f__Microbacteriaceae;g__Rhodoluna;s__* | 1 | *vanY gene in vanA cluster* | protein homolog model | glycopeptide | antibiotic target alteration | 32.32 | 79.21 |
| S3.bin12 | *d__Bacteria;p__Actinobacteriota;c__Acidimicrobiia;o__Acidimicrobiales;f__Ilumatobacteraceae;g__UBA3006;s__* | None |  |  |  |  |  |  |
| S3.bin13 | *d__Bacteria;p__Bacteroidota;c__Bacteroidia;o__Chitinophagales;f__Chitinophagaceae;g__JJ008;s__JJ008 sp005791465* | 1 | *vanT gene in vanG cluster* | protein homolog model | glycopeptide | antibiotic target alteration | 34.88 | 111.1 |
| S3.bin14 | *d__Bacteria;p__Actinobacteriota;c__Thermoleophilia;o__Solirubrobacterales;f__Solirubrobacteraceae;g__F1-60-MAGs163;s__* | None |  |  |  |  |  |  |
| S3.bin15 | *d__Bacteria;p__Proteobacteria;c__Gammaproteobacteria;o__Burkholderiales;f__Burkholderiaceae;g__DSNY01;s__* | None |  |  |  |  |  |  |
| S3.bin16 | *d__Bacteria;p__Proteobacteria;c__Gammaproteobacteria;o__Burkholderiales;f__Burkholderiaceae;g__Limnohabitans_A;s__* | None |  |  |  |  |  |  |
| S3.bin17 | *d__Bacteria;p__Planctomycetota;c__Planctomycetia;o__Pirellulales;f__UBA1268;g__QWPN01;s__* | 3 | *adeF* | protein homolog model | fluoroquinolone, tetracycline | antibiotic efflux | 47.64 | 100 |
|  |  |  | *qacG* | protein homolog model | disinfecting agents and antiseptics | antibiotic efflux | 44.34 | 101.87 |
|  |  |  | *adeF* | protein homolog model | fluoroquinolone, tetracycline | antibiotic efflux | 47.13 | 99.72 |
| S3.bin18 | *d__Bacteria;p__Proteobacteria;c__Gammaproteobacteria;o__Burkholderiales;f__Burkholderiaceae;g__Polaromonas;s__* | None |  |  |  |  |  |  |
| S3.bin19 | *d__Bacteria;p__Actinobacteriota;c__Acidimicrobiia;o__Acidimicrobiales;f__Ilumatobacteraceae;g__F1-20-MAGs119;s__* | 1 | *Mycobacterium tuberculosis rpsL mutations conferring resistance to Streptomycin* | protein variant model | aminoglycoside antibiotic | antibiotic target alteration | 85.37 | 99.19 |
| S3.bin20 | *d__Bacteria;p__Verrucomicrobiota;c__Verrucomicrobiae;o__Verrucomicrobiales;f__Akkermansiaceae;g__Luteolibacter;s__* | 2 | *vanT gene in vanG cluster* | protein homolog model | glycopeptide | antibiotic target alteration | 33.06 | 51.12 |
|  |  |  | *vanH gene in vanO cluster* | protein homolog model | glycopeptide | antibiotic target alteration | 37.62 | 97.14 |
| S3.bin21 | *d__Bacteria;p__Proteobacteria;c__Alphaproteobacteria;o__Sphingomonadales;f__Sphingomonadaceae;g__Sphingorhabdus_B;s__* | None |  |  |  |  |  |  |
| S3.bin22 | *d__Bacteria;p__Actinobacteriota;c__Acidimicrobiia;o__Acidimicrobiales;f__Ilumatobacteraceae;g__UBA2093;s__* | 2 | *vanT gene in vanG cluster* | protein homolog model | glycopeptide | antibiotic target alteration | 32.7 | 52.81 |
|  |  |  | *Mycobacterium tuberculosis rpsL mutations conferring resistance to Streptomycin* | protein variant model | aminoglycoside | antibiotic target alteration | 86.18 | 99.19 |
| S3.bin23 | *d__Bacteria;p__Proteobacteria;c__Gammaproteobacteria;o__Burkholderiales;f__Burkholderiaceae;g__Limnohabitans;s__Limnohabitans sp009923505* | None |  |  |  |  |  |  |
| S4.bin1 | *d__Bacteria;p__Actinobacteriota;c__Thermoleophilia;o__Solirubrobacterales;f__70-9;g__67-14;s__* | None |  |  |  |  |  |  |
| S4.bin2 | *d__Bacteria;p__Actinobacteriota;c__Acidimicrobiia;o__Acidimicrobiales;f__Ilumatobacteraceae;g__F1-60-MAGs027;s__F1-60-MAGs027 sp009926705* | None | *Mycobacterium tuberculosis rpsL mutations conferring resistance to Streptomycin* | protein variant model | aminoglycoside | antibiotic target alteration | 84.55 | 99.19 |
| S4.bin3 | *d__Bacteria;p__Proteobacteria;c__Gammaproteobacteria;o__Burkholderiales;f__Methylophilaceae;g__Methylopumilus;s__Methylopumilus rimovensis* | 1 | *adeF* | protein homolog model | fluoroquinolone, tetracycline | antibiotic efflux | 42.95 | 97.17 |
| S4.bin4 | *d__Bacteria;p__Actinobacteriota;c__Actinomycetia;o__Mycobacteriales;f__Mycobacteriaceae;g__Mycobacterium;s__* | 1 | *RbpA* | protein homolog model | rifamycin | antibiotic target protection | 93.69 | 97.37 |
|  |  |  | *vanY gene in vanB cluster* | protein homolog model | glycopeptide | antibiotic target alteration | 33.9 | 69.4 |
| S4.bin5 | *d__Bacteria;p__Verrucomicrobiota;c__Verrucomicrobiae;o__Chthoniobacterales;f__Terrimicrobiaceae;g__UBA967;s__* | 3 | *vanT gene in vanG cluster* | protein homolog model | glycopeptide | antibiotic target alteration | 31.88 | 51.69 |
|  |  |  | *qacG* | protein homolog model | disinfecting agents and antiseptics | antibiotic efflux | 42 | 100 |
|  |  |  | *adeF* | protein homolog model | fluoroquinolone, tetracycline | antibiotic efflux | 41.15 | 99.06 |
| S4.bin6 | *d__Bacteria;p__Chloroflexota;c__Anaerolineae;o__Caldilineales;f__Caldilineaceae;g__Caldilinea;s__Caldilinea sp018969295* | 2 | *vanW gene in vanI cluster* | protein homolog model | glycopeptide | antibiotic target alteration | 31.78 | 150.67 |
|  |  |  | *vanT gene in vanG cluster* | protein homolog model | glycopeptide | antibiotic target alteration | 35.52 | 54.63 |
| S4.bin7 | *d__Bacteria;p__Actinobacteriota;c__Thermoleophilia;o__Solirubrobacterales;f__70-9;g__67-14;s__* | None |  |  |  |  |  |  |
| S4.bin8 | *d__Bacteria;p__Planctomycetota;c__Planctomycetia;o__Pirellulales;f__UBA1268;g__QWPN01;s__* | 3 | *qacG* | protein homolog model | disinfecting agents and antiseptics | antibiotic efflux | 44.23 | 105.61 |
|  |  |  | *adeF* | protein homolog model | fluoroquinolone, tetracycline | antibiotic efflux | 47.09 | 95.85 |
|  |  |  | *vanH gene in vanO cluster* | protein homolog model | glycopeptide | antibiotic target alteration | 37.79 | 95.71 |
| S4.bin9 | *d__Bacteria;p__Actinobacteriota;c__Thermoleophilia;o__Solirubrobacterales;f__70-9;g__67-14;s__* | 1 | *vanT gene in vanG cluster* | protein homolog model | glycopeptide | antibiotic target alteration | 34.5 | 52.53 |
| S4.bin10 | *d__Bacteria;p__Proteobacteria;c__Alphaproteobacteria;o__Rhizobiales;f__Beijerinckiaceae;g__Methylocystis;s__Methylocystis sp002412985* | 2 | *adeF* | protein homolog model | fluoroquinolone, tetracycline | antibiotic efflux | 43.05 | 98.77 |
|  |  |  | *qacG* | protein homolog model | disinfecting agents and antiseptics | antibiotic efflux | 42.72 | 102.8 |
| S4.bin11 | *d__Bacteria;p__Cyanobacteria;c__Cyanobacteriia;o__Cyanobacteriales;f__Microcoleaceae;g__Planktothrix;s__Planktothrix agardhii* | 4 | *vanY gene in vanM cluster* | protein homolog model | glycopeptide | antibiotic target alteration | 34.35 | 124.46 |
|  |  |  | *vanT gene in vanG cluster* | protein homolog model | glycopeptide | antibiotic target alteration | 34.76 | 56.32 |
|  |  |  | *vanH gene in vanB cluster* | protein homolog model | glycopeptide | antibiotic target alteration | 39.93 | 106.5 |
|  |  |  | *qacJ* | protein homolog model | disinfecting agents and antiseptics | antibiotic efflux | 39.8 | 100 |
| S4.bin12 | *d__Bacteria;p__Actinobacteriota;c__Acidimicrobiia;o__Acidimicrobiales;f__Ilumatobacteraceae;g__UBA3006;s__* | None |  |  |  |  |  |  |
| S4.bin13 | *d__Bacteria;p__Actinobacteriota;c__Acidimicrobiia;o__Acidimicrobiales;f__Ilumatobacteraceae;g__F1-60-MAGs027;s__* | 2 | *Mycobacterium tuberculosis rpsL mutations conferring resistance to Streptomycin* | protein variant model | aminoglycoside | antibiotic target alteration | 86.07 | 98.39 |
|  |  |  | *Mycobacterium tuberculosis rpsL mutations conferring resistance to Streptomycin* | protein variant model | aminoglycoside | antibiotic target alteration | 84.55 | 99.19 |
| S4.bin14 | *d__Bacteria;p__Planctomycetota;c__Planctomycetia;o__Pirellulales;f__UBA1268;g__QWPN01;s__* | 5 | *adeF* | protein homolog model | fluoroquinolone, tetracycline | antibiotic efflux | 48.27 | 99.62 |
|  |  |  | *qacG* | protein homolog model | disinfecting agents and antiseptics | antibiotic efflux | 48.11 | 101.87 |
|  |  |  | *adeF* | protein homolog model | fluoroquinolone, tetracycline | antibiotic efflux | 45.73 | 89.33 |
|  |  |  | *adeF* | protein homolog model | fluoroquinolone, tetracycline | antibiotic efflux | 47.72 | 100.09 |
|  |  |  | *qacG* | protein homolog model | disinfecting agents and antiseptics | antibiotic efflux | 37.74 | 101.87 |
| S4.bin15 | *d__Bacteria;p__Planctomycetota;c__Planctomycetia;o__Pirellulales;f__UBA1268;g__RGVT01;s__* | 1 | *qacG* | protein homolog model | disinfecting agents and antiseptics | antibiotic efflux | 43.4 | 101.87 |
| S4.bin16 | *d__Bacteria;p__Actinobacteriota;c__Thermoleophilia;o__Solirubrobacterales;f__Solirubrobacteraceae;g__F1-60-MAGs163;s__* | None |  |  |  |  |  |  |
| S5.bin1 | *d__Bacteria;p__Proteobacteria;c__Gammaproteobacteria;o__Xanthomonadales;f__Xanthomonadaceae;g__Arenimonas;s__* | None |  |  |  |  |  |  |
| S5.bin2 | *d__Bacteria;p__Proteobacteria;c__Gammaproteobacteria;o__Burkholderiales;f__Burkholderiaceae;g__Limnohabitans_A;s__* | None |  |  |  |  |  |  |
| S5.bin3 | *d__Bacteria;p__Planctomycetota;c__Planctomycetia;o__Pirellulales;f__Pirellulaceae;g__Pirellula_B;s__* | 2 | *qacG* | protein homolog model | disinfecting agents and antiseptics | antibiotic efflux | 42.45 | 103.74 |
|  |  |  | *adeF* | protein homolog model | fluoroquinolone, tetracycline | antibiotic efflux | 48.95 | 100.09 |
| S5.bin4 | *d__Bacteria;p__Actinobacteriota;c__Actinomycetia;o__Nanopelagicales;f__Nanopelagicaceae;g__MAG-120802;s__MAG-120802 sp018969585* | None |  |  |  |  |  |  |
| S5.bin5 | *d__Bacteria;p__Bacteroidota;c__Bacteroidia;o__Chitinophagales;f__Chitinophagaceae;g__JJ008;s__JJ008 sp005791465* | 1 | *vanT gene in vanG cluster* | protein homolog model | glycopeptide | antibiotic target alteration | 34.88 | 111.10 |
| S5.bin6 | *d__Bacteria;p__Actinobacteriota;c__Acidimicrobiia;o__Acidimicrobiales;f__Ilumatobacteraceae;g__Casp-actino8;s__* | 2 | *vanT gene in vanG cluster* | protein homolog model | glycopeptide | antibiotic target alteration | 33.51 | 53.23 |
|  |  |  | *Mycobacterium tuberculosis rpsL mutations conferring resistance to Streptomycin* | protein variant model | aminoglycoside | antibiotic target alteration | 85.37 | 99.19 |
| S5.bin7 | *d__Bacteria;p__Cyanobacteria;c__Cyanobacteriia;o__Cyanobacteriales;f__Microcystaceae;g__Microcystis;s__Microcystis aeruginosa* | 2 | *vanY gene in vanM cluster* | protein homolog model | glycopeptide | antibiotic target alteration | 34.33 | 84.98 |
|  |  |  | *vanY gene in vanA cluster* | protein homolog model | glycopeptide | antibiotic target alteration | 39.51 | 38.28 |
| S5.bin8 | *d__Bacteria;p__Actinobacteriota;c__Acidimicrobiia;o__Acidimicrobiales;f__Ilumatobacteraceae;g__UBA3006;s__* | 1 | *Mycobacterium tuberculosis rpsL mutations conferring resistance to Streptomycin* | protein variant model | aminoglycoside | antibiotic target alteration | 86.07 | 98.39 |
| S5.bin9 | *d__Bacteria;p__Bacteroidota;c__Bacteroidia;o__NS11-12g;f__UBA955;g__UBA955;s__UBA955 sp002293105* | None |  |  |  |  |  |  |
| S5.bin10 | *d__Bacteria;p__Actinobacteriota;c__Acidimicrobiia;o__Acidimicrobiales;f__Ilumatobacteraceae;g__BACL27;s__* | 1 | *vanT gene in vanG cluster* | protein homolog model | glycopeptide | antibiotic target alteration | 34.88 | 52.95 |
| S5.bin11 | *d__Bacteria;p__Gemmatimonadota;c__Gemmatimonadetes;o__Gemmatimonadales;f__Gemmatimonadaceae;g__PNKF01;s__* | None |  |  |  |  |  |  |
| S5.bin12 | *d__Bacteria;p__Proteobacteria;c__Alphaproteobacteria;o__Pelagibacterales;f__Pelagibacteraceae;g__Fonsibacter;s__Fonsibacter ubiquis* | None |  |  |  |  |  |  |
| S6.bin1 | *d__Bacteria;p__Proteobacteria;c__Alphaproteobacteria;o__Rhizobiales;f__Beijerinckiaceae;g__Methylocystis;s__Methylocystis sp002412985* | 2 | *qacG* | protein homolog model | disinfecting agents and antiseptics | antibiotic efflux | 42.72 | 102.8 |
| S6.bin2 | *d__Bacteria;p__Actinobacteriota;c__Acidimicrobiia;o__Acidimicrobiales;f__Ilumatobacteraceae;g__VFMC01;s__* | 1 | *Mycobacterium tuberculosis rpsL mutations conferring resistance to Streptomycin* | protein variant model | aminoglycoside | antibiotic target alteration | 85.37 | 99.19 |
| S6.bin3 | *d__Bacteria;p__Actinobacteriota;c__Acidimicrobiia;o__Acidimicrobiales;f__Ilumatobacteraceae;g__UBA3006;s__* | 1 | *Mycobacterium tuberculosis rpsL mutations conferring resistance to Streptomycin* | protein variant model | aminoglycoside | antibiotic target alteration | 83.61 | 98.39 |
| S6.bin4 | *d__Bacteria;p__Verrucomicrobiota;c__Verrucomicrobiae;o__Chthoniobacterales;f__UBA6821;g__UBA6821;s__* | 2 | *vanH gene in vanB cluster* | protein homolog model | glycopeptide | antibiotic target alteration | 37.98 | 109.91 |
|  |  |  | *adeF* | protein homolog model | fluoroquinolone, tetracycline | antibiotic efflux | 41.89 | 96.41 |
| S6.bin5 | *d__Bacteria;p__Cyanobacteria;c__Cyanobacteriia;o__Cyanobacteriales;f__Microcoleaceae;g__Planktothrix;s__Planktothrix agardhii* | 4 | *vanT gene in vanG cluster* | protein homolog model | glycopeptide | antibiotic target alteration | 34.76 | 56.32 |
|  |  |  | *qacJ* | protein homolog model | disinfecting agents and antiseptics | antibiotic efflux | 39.8 | 100 |
|  |  |  | *vanY gene in vanM cluster* | protein homolog model | glycopeptide | antibiotic target alteration | 34.35 | 124.46 |
|  |  |  | *vanH gene in vanB cluster* | protein homolog model | glycopeptide | antibiotic target alteration | 39.93 | 103.41 |
| S6.bin6 | *d__Bacteria;p__Actinobacteriota;c__Thermoleophilia;o__Solirubrobacterales;f__Solirubrobacteraceae;g__F1-60-MAGs163;s__* | None |  |  |  |  |  |  |
| S6.bin7 | *d__Bacteria;p__Planctomycetota;c__Planctomycetia;o__Pirellulales;f__UBA1268;g__QWPN01;s__* | 2 | *adeF* | protein homolog model | fluoroquinolone, tetracycline | antibiotic efflux | 48.27 | 99.62 |
|  |  |  | *qacG* | protein homolog model | disinfecting agents and antiseptics | antibiotic efflux | 48.11 | 101.87 |
| S6.bin8 | *d__Bacteria;p__Chloroflexota;c__Anaerolineae;o__Caldilineales;f__Caldilineaceae;g__Caldilinea;s__* | 1 | *vanW gene in vanI cluster* | protein homolog model | glycopeptide | antibiotic target alteration | 31.78 | 150.67 |
| S6.bin9 | *d__Bacteria;p__Bacteroidota;c__Kapabacteria;o__Kapabacteriales;f__UBA961;g__UBA961;s__* | None |  |  |  |  |  |  |
| S6.bin10 | *d__Bacteria;p__Verrucomicrobiota;c__Verrucomicrobiae;o__Chthoniobacterales;f__JAATET01;g__;s__* | None |  |  |  |  |  |  |
| S6.bin11 | *d__Bacteria;p__Planctomycetota;c__Planctomycetia;o__Pirellulales;f__UBA1268;g__RGVT01;s__* | 1 | *qacG* | protein homolog model | disinfecting agents and antiseptics | antibiotic efflux | 43.4 | 101.87 |
| S6.bin12 | *d__Bacteria;p__Verrucomicrobiota;c__Verrucomicrobiae;o__Chthoniobacterales;f__Terrimicrobiaceae;g__UBA967;s__* | 3 | *qacG* | protein homolog model | disinfecting agents and antiseptics | antibiotic efflux | 42 | 100 |
|  |  |  | *vanT gene in vanG cluster* | protein homolog model | glycopeptide | antibiotic target alteration | 31.88 | 51.69 |
|  |  |  | *adeF* | protein homolog model | fluoroquinolone, tetracycline | antibiotic efflux | 41.15 | 99.06 |
| S6.bin13 | *d__Bacteria;p__Proteobacteria;c__Alphaproteobacteria;o__Pelagibacterales;f__Pelagibacteraceae;g__Fonsibacter;s__Fonsibacter sp018882565* | None |  |  |  |  |  |  |

**Table S12.** Top BLAST results for select ARGs annotated on Cyanobacteria contigs/MAGs.

| ARG hit | Tool | CAT or GTDBK Taxonomy | Contig/Bin | Sample | BLAST method | Relevant BLAST Results | Query cover | E value | Per. Ident | Accession |
| --- | --- | --- | --- | --- | --- | --- | --- | --- | --- | --- |
| *vanY in vanM cluster* | RGI | *Planktothrix agardhii* | k99_274016; bin3 | GLSM2 | BLASTp | M15 family metallopeptidase [Planktothrix agardhii] | 100% | 0 | 100 | WP_081846422.1 |
| *qacJ* | RGI | *Planktothrix agardhii* | k99_496202; bin3 | GLSM2 | BLASTp | multidrug efflux SMR transporter [Planktothrix] | 100% | 9.00E-66 | 100 | WP_042154449.1 |
|  |  |  |  |  | BLASTp | TPA: hypothetical protein [Planktothrix sp. UBA8407] | 100% | 1.00E-64 | 98.13 | HAO11831.1 |
|  |  |  |  |  | BLASTp | multidrug efflux SMR transporter [Planktothrix agardhii KL2] | 97% | 6.00E-63 | 99.04 | MBG0745825.1 |
| *arlR* | DeepARG | *Microcystis* | k99_1358475 | LakeErie3 | BLASTn | Microcystis aeruginosa NRERC-214 chromosome, complete genome; response regulator transcription factor | 100% | 0 | 98.57 | CP134413.1 |
|  |  |  |  |  | BLASTn | Microcystis aeruginosa PCC 7806SL chromosome, complete genome | 97% | 0 | 97.62 | CP020771.1 |
| *rpoB2* | DeepARG | *no support* | k99_952662 | LakeErie3 | BLASTn | Microcystis aeruginosa FD4 chromosome, complete genome; DNA-directed RNA polymerase subunit beta | 100% | 0 | 96.45 | CP046973.1 |
|  |  |  |  |  | BLASTn | Microcystis viridis NIES-102 DNA, complete genome; RNA polymerase beta subunit | 100% | 0 | 97.04 | AP019314.1 |
| *vatB* | DeepARG | *Pseudanabaena* | k99_752142 | LakeErie3 | BLASTn | Pseudanabaena sp. ABRG5-3 DNA, complete genome; ann O-acetyltransferase | 39% | 0 | 89.78 | AP017560.1 |
|  |  |  |  |  | BLASTn | Pseudanabaena sp. Chao 1811 chromosome, complete genome | 39% | 0 | 89.42 | CP101416.1 |
|  |  |  |  |  | BLASTn | Pseudanabaena galeata CCNP1313 chromosome, complete genome; Vat family streptogramin A O-acetyltransferase | 38% | 3.00E-175 | 86.28 | CP112874.1 |
| OXA | DeepARG | *Synechococcus* | k99_1036155 | LakeErie3 | BLASTn | Synechococcus sp. RS9909 chromosome, complete genome; penicillin binding transpeptidase domain protein | 94% | 0 | 92.22 | CP047943.1 |
|  |  |  |  |  | BLASTp with gene from top BLASTn hit | class D beta-lactamase [unclassified Synechococcus] | 100% | 0 | 100 | WP_007100094.1 |
|  |  |  |  |  | BLASTp with gene from top BLASTn hit | class D beta-lactamase [Synechococcus sp. XM-24] | 95% | 2.00E-146 | 78.85 | PWL23196.1 |
|  |  |  |  |  | BLASTp with gene from top BLASTn hit | class D beta-lactamase [Synechococcus sp. WH 8101] | 94% | 3.00E-146 | 80.08 | WP_165380923.1 |
| *EmrE* | DeepARG | *Cyanobium* | k99_956387 | LakErie5 | BLASTn | Cyanobium gracile PCC 6307, complete genome; putative flavoprotein | 47% | 0 | 91.07 | CP003495.1 |
|  |  |  |  |  | BLASTn | Synechococcus sp. CBW1107 chromosome, complete genome; MBL fold metallo-hydrolase | 46% | 6.00E-121 | 74.5 | CP064908.1 |

**Table S13.** Insertion Sequences Annotated on Cyanobacteria MAGs.

| **Bin name(s)** | **Lowest taxonomy classification** | **MGE No.** | **Name** | **Type** | **Identity** | **Coverage** |
| --- | --- | --- | --- | --- | --- | --- |
| S2.bin2, S4.bin11, S6.bin5 | *s__Planktothrix agardhii* | 1 | ISMae11 | Insertion sequence | 0.744666 | 0.909339 |
|  |  | 2 | ISPlag1 | Insertion sequence | 0.988868 | 0.412711 |
|  |  | 3 | ISPasp1 | Insertion sequence | 0.755245 | 0.134578 |
|  |  | 4 | ISMae41 | Insertion sequence | 0.741598 | 0.961393 |
|  |  | 5 | ISAsp18 | Insertion sequence | 0.821718 | 0.679021 |
|  |  | 6 | ISMae41 | Insertion sequence | 0.86188 | 0.794852 |
|  |  | 7 | ISMae6 | Insertion sequence | 0.78777 | 0.159334 |
|  |  | 8 | ISAsp18 | Insertion sequence | 0.862884 | 0.281818 |
|  |  | 9 | ISPlr1 | Insertion sequence | 0.799197 | 0.164441 |
|  |  | 10 | ISAsp12 | Insertion sequence | 0.782251 | 0.773535 |
| S3.bin3 | *g__Cyanobium* | 1 | ISTsu2 | Insertion sequence | 0.728395 | 0.143312 |
| S5.bin7 | *s__Microcystis aeruginosa* | 1 | ISMae39 | Insertion sequence | 0.901316 | 0.275641 |
|  |  | 2 | ISMae11 | Insertion sequence | 0.895582 | 0.163599 |
|  |  | 3 | ISMae14 | Insertion sequence | 1 | 0.104685 |
|  |  | 4 | ISMae15 | Insertion sequence | 0.926302 | 0.481346 |
|  |  | 5 | ISMae15 | Insertion sequence | 0.85782 | 0.129052 |
|  |  | 6 | ISMae8 | Insertion sequence | 0.971609 | 0.223554 |
|  |  | 7 | ISMae43 | Insertion sequence | 0.93865 | 0.12916 |
|  |  | 8 | ISCysp6 | Insertion sequence | 0.787234 | 0.101471 |
|  |  | 9 | ISMae22 | Insertion sequence | 0.950867 | 0.303163 |
|  |  | 10 | ISMae41 | Insertion sequence | 0.794964 | 0.196064 |
|  |  | 11 | ISMae8 | Insertion sequence | 0.959707 | 0.192525 |
|  |  | 12 | ISMae27 | Insertion sequence | 0.732839 | 0.923818 |
|  |  | 13 | ISMae16 | Insertion sequence | 0.925501 | 0.239886 |
|  |  | 14 | ISMae13 | Insertion sequence | 0.797414 | 0.623686 |
|  |  | 15 | ISMae34 | Insertion sequence | 0.891892 | 0.108584 |
|  |  | 16 | ISMae11 | Insertion sequence | 0.961616 | 0.337423 |
|  |  | 17 | ISMae13 | Insertion sequence | 0.920564 | 0.510161 |
|  |  | 18 | ISMae16 | Insertion sequence | 0.978261 | 0.225692 |
|  |  | 19 | ISMae4 | Insertion sequence | 0.95122 | 0.134647 |
|  |  | 20 | ISMae41 | Insertion sequence | 0.937888 | 0.121877 |
|  |  | 21 | ISMae28 | Insertion sequence | 0.927273 | 0.103448 |
|  |  | 22 | ISMae41 | Insertion sequence | 0.947977 | 0.130961 |
|  |  | 23 | ISMae41 | Insertion sequence | 0.980263 | 0.11355 |
|  |  | 24 | ISMae25 | Insertion sequence | 0.989474 | 0.152488 |
|  |  | 25 | ISMae10 | Insertion sequence | 0.843373 | 0.116257 |
|  |  | 26 | ISMae11 | Insertion sequence | 0.917068 | 0.697342 |
|  |  | 27 | ISMae39 | Insertion sequence | 0.867403 | 0.165751 |
|  |  | 28 | ISMae11 | Insertion sequence | 0.883534 | 0.163599 |
|  |  | 29 | ISMae39 | Insertion sequence | 0.88785 | 0.184982 |
